# Natural Products of Plants and Animal Origin Improve Albumen Quality of Chicken Eggs

**DOI:** 10.3389/fnut.2022.875270

**Published:** 2022-06-10

**Authors:** Uchechukwu Edna Obianwuna, Vivian U. Oleforuh-Okoleh, Jing Wang, Hai-Jun Zhang, Guang-Hai Qi, Kai Qiu, Shu-Geng Wu

**Affiliations:** ^1^National Engineering Research Center of Biological Feed, Feed Research Institute, Chinese Academy of Agricultural Sciences, Beijing, China; ^2^Department of Animal Science, Faculty of Agriculture, Rivers State University, Port Harcourt, Nigeria

**Keywords:** albumen quality, natural feed additives, laying hens, safe product, high-quality eggs

## Abstract

Albumen quality is recognized as one of the major yardsticks in measuring egg quality. The elasticity of thick albumen, a strong bond in the ovomucin-lysozyme complex, and excellent biological properties are indicators of high-quality albumen. The albumen quality prior to egg storage contribute to enhance egg’s shelf life and economic value. Evidence suggests that albumen quality can deteriorate due to changes in albumen structure, such as the degradation of β-ovomucin subunit and *O*-glyosidic bonds, the collapse of the ovomucin-lysozyme complex, and a decrease in albumen protein-protein interaction. Using organic minerals, natural plants and animal products with antioxidant and antimicrobial properties, high biological value, no residue effect and toxicity risk could improve albumen quality. These natural products (e.g., tea polyphenols, marigold extract, magnolol, essential oils, Upro (small peptide), yeast cell wall, *Bacillus* species, a purified amino acid from animal blood, and pumpkin seed meal) are bio-fortified into eggs, thus enhancing the biological and technological function of the albumen. Multiple strategies to meeting laying hens’ metabolic requirements and improvement in albumen quality are described in this review, including the use of amino acids, vitamins, minerals, essential oils, prebiotics, probiotics, organic trace elements, and phytogenic as feed additives. From this analysis, natural products can improve animal health and consequently albumen quality. Future research should focus on effects of these natural products in extending shelf life of the albumen during storage and at different storage conditions. Research in that direction may provide insight into albumen quality and its biological value in fresh and stored eggs.

## Introduction

Globally, meeting the demands of an ever-increasing population and per catput protein requirement is quite a challenge. Chicken eggs are among the most commonly consumed animal protein worldwide due to their low cost, nutritional contents, and biological functions (1), thus significantly contributing to human nutrition. Rapid growth in poultry production has reflected in direct increase in egg production. Critical concern in egg production is the issue of maintenance of egg quality, particularly the albumen quality, since most of the functional properties of the egg is related to the albumen.

The albumen contains proteins (ovomucin, ovalbumin, ovotransferrin, and lysozyme), peptides and amino acids which are natural antioxidant compounds (2). The ovomucin is considered as one of the core albumen proteins that plays a key role in maintaining albumen structure by stabilizing protein-protein bond interaction, viscous-gel nature, and height of the thick albumen, culminating in increased Haugh unit value (3, 4). The Haugh unit (HU) value is often used to measure albumen quality, high HU value depicts thick albumen content and gelly nature with strong viscosity which reflects better albumen quality while low HU unit value is vice versa (5). The improved albumen structure and proteins, confers on the albumen its biological functions and excellent technological properties such as foaming, gelling, emulsifying and water holding capacity. Albumen of improved quality could be utilized as raw material for food processing and health industry; consumption of food-derived antioxidants such as egg white peptides and egg white powder as functional foods exert beneficial effects on human health conditions by scavenging reactive oxygen species and free-radical mediated chain reactions (6). On the other hand, poor albumen quality is probably due to reduced ovomucin-lysozyme complex interaction, disaggregation of ovomucin subunits (α- and β-) and reduction of highly glycosylated β- subunit in thick albumen gel (3, 7, 8). All these changes eventually collapses gelatinous structure of thick albumen into a clear liquid (albumen thinning) as albumen consistency is lost (9) as well as the rheological, nutritional and technological properties which consumers rely on. Therefore, exploring the potentials of egg albumen as functional food and ingredient is reliant on the quality of albumen (technological and biological properties) obtained from the egg.

Albumen quality are influenced by various factors; animal feed (10), birds age (11) and storage environment (12). The role of diets in animal health and consequently performance cannot be overemphasized; cottonseed meal reduced albumen proteins and viscosity, and impaired ovary health (13), vanadium, an inorganic mineral, caused cell apoptosis in the magnum and reduced the immune capacity function of laying hens (10) while dietary fluoride reduced albumen quality and immune function of the animals (14). Therefore, adoption of nutritional modulations as a strategy for improving albumen quality and animal health is advocated for.

However, the use of non-natural feed source is often associated with challenges such as antinutritional factors, accurate inclusion levels, negative effect on animal health (15), and consumer concerns about residue, toxicity, pollution, and low biological value limit their optimal use. In recent times, the use of natural products from plants and animal origin as feed additives in diets of laying hens have gained attention. The use of natural-source feed such as natural products with antioxidant and antimicrobial properties derived from plants and animals are advantageous because they pose no residue effect, pollution, or toxicity risk. Natural products including antimicrobial peptides (16), purified amino acids from animal blood (17), small peptide synthesized from corn (18), prebiotics (19), probiotics (20), essential oil (21) and organic trace elements in the form of chelates or amino acid complexes (22) have been reported to influence albumen quality. The improvement in albumen quality with natural products are associated with their high bioavailability, antioxidant function and capacity to maintain gut microflora, which is important for animal health and bio-fortification of animal products. The improved albumen quality is important to both consumers and producers. As a result, this review provides an in-depth understanding of the impact of various natural diets of plant and animal origin and their inclusion levels on egg albumen quality and overall health of laying hens.

## Overview of Albumen Quality

### Albumen Structure and Composition

**FIGURE 1:**
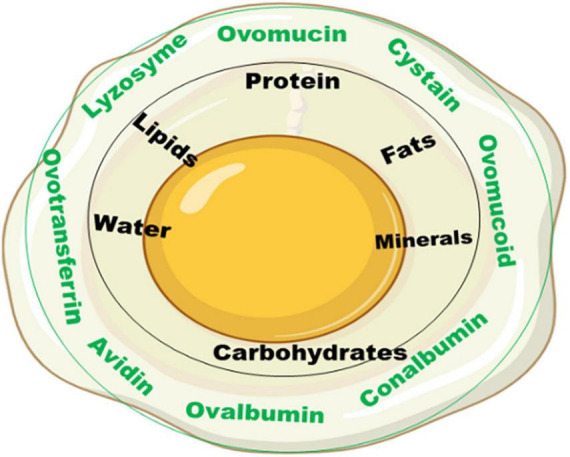
Albumen structure and its composition.

Egg albumen is a homogeneous, non-crystalline substance (liquid) formed around the egg yolk (Figure 1). The albumen structurally is composed of different layers, each with its peculiar function, namely: the chalaziferous white or inner thick layer resting around the yolk which stabilizes the yolk movement such that it maintains its center position; the inner thin layer lying very close to the chalazae which serves as a protecting capsule enveloping the yolk (this layer has more fluid and contains most of the albumen protein); the outer thick white layer, lying adjacent to the inner thin white layer, provides further fluid and texture to the albumen fluids; and the outer thin white layer which holds further protein-based nutrients and compounds that play essential role in the growth and development of embryo in the fertilized egg (23, 24). The principal function of the albumen is as a natural defense system for the yolk in table eggs and embryo in fertile eggs.

**FIGURE 2:**
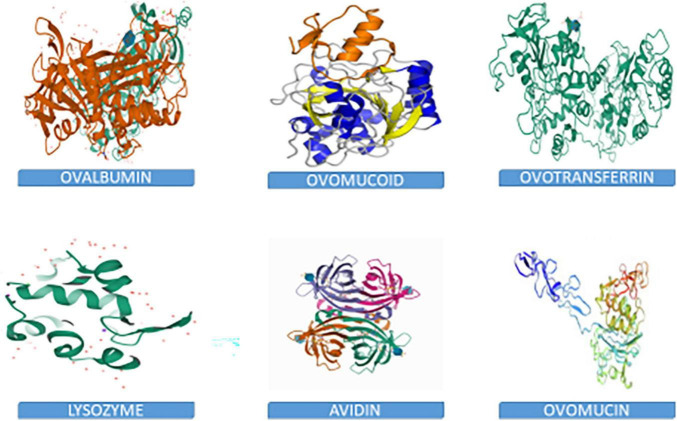
3D structures of albumen proteins.

**FIGURE 3:**
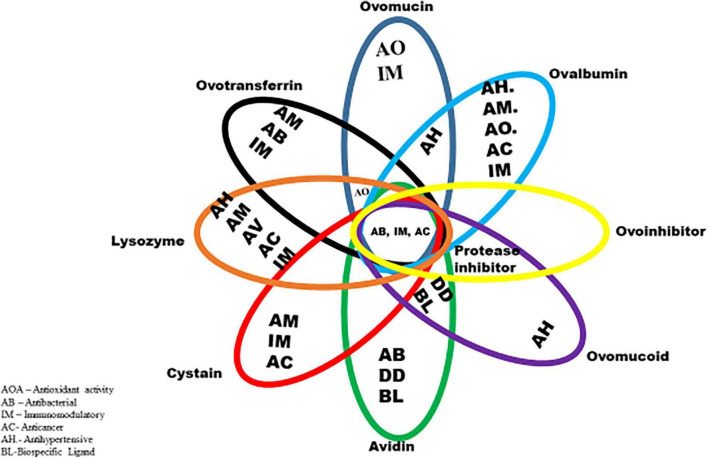
Biological functions of albumen proteins.

Chemically, the albumen consists primarily of water and proteins, containing about 88% water, and 10–11% proteins. Other nutrients such as trace minerals, vitamins, lipids and carbohydrates are also found in the albumen. The albumen proteins are, however, of much more biological relevance than other nutrients. The major albumen proteins include ovalbumin, ovomucoid, conalbumin, ovomucin and lysozyme (Figure 2). The ovoglobulins, ovomaroglobulin, avidin, ovoinhibitor, cystatin, ovoglycoprotein, and ovoflavoprotein are considered minor albumen proteins (23). However, each of the albumen proteins has specific physical and chemical characteristics which confer critical biological functions on the albumen, as shown in Figure 3.

The foaming (whippability), gelling (coagulation) and emulsifying properties are conferred on the albumen by (ovalbumin, lysozyme, and ovomucin); antimicrobial properties (lysozyme and ovotransferrin), which allows its exploitation as a functional food material in many food, pharmaceuticals and other material industries (24). The mechanisms through which these proteins exert biological functions, may include bacterial cell wall degradation and binding to vitamins and metals (25). Of all the different types of protein found in the egg albumen, the ovomucin content has been demonstrated by various studies to, play a significant role in determining the albumen nature and hence its quality (3, 8, 26, 27).

### The Ovomucin Structure and Relationship With Albumen Quality

Ovomucin is a glycoprotein accounting for nearly 3.5% of total egg white protein. It is made up of α- and β-subunits with molecular weights of 5.5 and 8.3, and isoelectric points (pI) of 4 and 5.5, respectively (3). α-ovomucin is homogenous and has lower carbohydrate content (15%), whereas β-ovomucin is heterogeneous and rich in carbohydrate (50%) (26). The carbohydrate chain of ovomucin consists of sialic acid (2.5–8%), hexose (15–18.6%), and hexosamine (7–12%) (6). The amino acid content of the two ovomucin subunits differs: α- and β-subunits of the ovomucin, respectively, contain acidic (glutamic acid and aspartic acid) and hydroxyl amino acids (threonine and serine) (28). Albumen’s gel-like properties are due to the complex formed by the two subunits of ovomucin, resulting in insoluble thick albumen (29). Ovomucin is strongly heat-stable because of its random coil nature, and, as such, it does not exhibit thermal transition temperature. Albumen liquefaction occurs when the α- and β-subunits, making up the thick albumen, are depolarized. This result in a lower proportion of insoluble ovomucin with a high carbohydrate content (26). There is a strong relationship between ovomucin and albumen quality because the β-ovomucin is fundamental to albumen quality determination.

It has been reported that ovomucin polymers and the ovomucin-lysozyme complex interaction are fundamental to albumen height, viscosity, elasticity, and the gelatinous nature (30). The ovomucin content and height of thick albumen are interrelated reflecting in the HU value, because of the relationship between HU value and ovomucin abundance (9). In addition, the thick albumen of eggs with high HU values had strongly glycosylated β-ovomucin compared to eggs with low HU values (27). A thick gelatinous nature, strong viscoelasticity, rich glycosylated β-ovomucin, and high HU values are albumen quality indicators largely driven by ovomucin content and activity. Improved thick albumen quality due to ovomucin content is important for producers, consumers, and breeders.

The ovomucin-albumen quality relationship is influenced by various factors, including; magnum morphology, the rate of albumen secretion, diets, storage time, and temperature. The β-ovomucin secreted by the magnum maintains magnum health (4), viscoelasticity of albumen (9), and increases albumen durability due to complex formation with proteins and polysaccharides (31). Hence, the quality of β-ovomucin secreted by the magnum indicates albumen quality. The incomplete mucosal columnar epithelial cell layer of the magnum and low magnum index reduces rate of ovomucin deposition in the albumen, thereby decreasing albumen secretion (32). Antinutritional factors impair protein digestion and secretion, thus reducing albumen secretion and ovomucin deposition in the albumen (33). Diets such as amino acids (28, 34) and green tea (4) enhanced β-ovomucin content in thick albumen while cotton seed meal (CSM) decreased ovomucin content (13). Increased storage time decreased ovomucin content in the albumen of stored eggs (35). The mechanism of how other albumen proteins contribute to albumen quality requires further investigation. Freshness of eggs is often assessed based on HU value and there exists a relationship between ovomucin abundance and HU value, thus HU value is a measure of albumen quality.

### Haugh Unit as an Indicator of Albumen Quality

The HU measures egg quality and stability based on the height of thick albumen and the egg’s weight (36). Also, the HU value is determined by the stability of the protein chains in the albumen. The viscous-gelly nature of the thick albumen reflected in the HU value is influenced by the ovomucin content (3). A strong correlation between the two indices (i.e., thick albumen and HU) was reported by Wang et al. (9). In addition, (8) found that birds of genetic lines with high HU scores had higher ovomucin content and isoleucine levels than those with low HU scores. The USDA used HU to classify egg quality as; AA-grade at HU > 72; A-grade at HU = 60–72; B-grade at HU < 60, C-grade < 31 (37), and high HU values indicate high albumen quality. The effects of various diets on HU values are presented in Supplementary Table 1. From the analysis: green tea, organic trace elements, phytobiotics and natural oil significantly improved HU values compared to other diets.

## Beneficial Effects of Albumen on Human Health and the Food Industry

Egg white proteins are excellent sources of bioactive peptides; thus, albumen of high quality is critical to the food and health sectors. Over the past decades, most bioactive peptides were derived from milk and soybean proteins, but peptides from egg white proteins have been produced recently. These food protein-derived bioactive peptides, are health enhancers and have quite less safety risk. Food bioactive peptides which exert physiological effects on humans, are inactive within the sequence of their parent protein and can be activated by enzymatic hydrolysis during food processing (38). Under different conditions, these active egg white peptides have been hydrolyzed with pepsin, trypsin, and α-chymotrypsin enzymes. The hydrolysis of egg white enhances the activities (antioxidant, antimicrobial, anticarcinogenic, mineral-binding, and antihypertensive) of albumen proteins and their peptides (39), confirming the multi-functionality of these egg-derived peptides. Egg white derived-bioactive peptides could be considered as natural antioxidants owing to its capacity to control lipid peroxidation and supply essential amino acids, thus have promising potential over synthetic antioxidants in the food-processing industry. Also, egg white bioactive peptides can be consumed as functional foods to replace synthetic antioxidants without dosage or safety concerns.

The reports of Manso et al. (40), Rupa et al. (41), Sun et al. (42), Garcés-Rimón et al. (43) demonstrated that functional peptides from egg white and various egg white proteins have beneficial effects on human health and the food industry; hence, they could be used as drugs and functional foods. Hydrolysis of albumen proteins yields peptides with strong antioxidant capacity. Alcalase peptide with the sequence (Tyr-Ala-Glu-Glu-Arg-Tyr-Pro-Ile-Leu) has a high antioxidant capacity and free radical scavenging ability (21, 43). In addition, peptides with the sequence (Try-Leu-Gly-Ala and Lys) and (Gly-Gly-Leu-Glu-Pro-Ile-Phe-Glu) exhibited significant antioxidant effects in 2,2-diphenyl-1-picrylhydrazyl radical scavenging capacity (DPPH) and lipid peroxidation inhibition assays (44). Moreover, (45) identified another peptide showing higher antioxidant capacity in oxygen radical absorbance capacity fluorescein (ORAC-FL) assay. The high-antioxidant nature of these egg white peptides harnesses their potentials to be consumed or used in food processing as natural food antioxidants.

The angiotensin-converting enzyme “ACE” suppressing activity of peptides promotes their use for mitigating high blood pressure. Ovalbumin peptides exert a high inhibitory effect on ACE activity (46). Chiang et al. (47) discovered that a bioactive peptide produced by hydrolyzing egg white protein with thermolysin inhibited ACE activity in hypertensive rats. Similarly, egg white peptides ameliorated oxidative stress and hyperlipidemia in hypertensive rats (40). Also, Garcés-Rimón et al. (43) demonstrated that bioactive peptides of egg white exerted strong antioxidant effects leading to prevention of abnormalities in experimental models that are obese and hypertensive. On the other hand, enzymatic hydrolysate of lysozyme produced LPH2, showing a strong ACE inhibitory effect, high antioxidant activity in ORAC-Fl assay, and resistance to gram-negative and gram-positive bacteria (48). These egg white derived bioactive peptides as natural products would provide a safe alternative to hypertension therapy over synthetic agents that may be associated with side effects.

Egg white proteins and its peptide derivatives have immunomodulatory properties, which allows them to be used to improve immune responses for cancer and other diseases. Egg white peptides can stimulate macrophage activity and block the tumor necrosis factor (TNF)-mediated NF-κB pathway (42), while tumor growth suppressions are linked to ovomucin glycopeptides (49). In addition, cystatin induces TNF and interleukin (IL)-10 synthesis while avidin enhances antitumor effect *via* improved upregulation of TNF-α and thus acts as an anticancer agent and drug carrier (50). Also, feeding ovalbumin peptides to hypertensive rats reduced blood pressure and had an immunomodulatory effect by increasing the production of anti-inflammatory cytokines (IL-12, 227 IL-17, and IL-10) (40), while inhibiting the production of IL-4 (41). Ovomucin and ovomucin-derived peptides induce macrophage synthesis *in vitro*, making them immune modulators (25). Ovotransferrin peptides have been shown to exert anticancer effect by improving the activity of superoxide dismutase and resistance of human breast and colon cells to cancer (51). Also, ovotransferrin peptides can form complexes easily with iron, and the bound iron can be in a free form at a pH of < 4.5 (52). Hence, ovotransferrin peptides can be used as human drugs in the form of iron supplements (53). This means they are useful in human medicine. It has been reported that feeding ovalbumin peptides to hypertensive rats reduced blood pressure and had an immunomodulatory effect by increasing the production of anti-inflammatory cytokines (40). Also, peptides of lysozyme stimulate immunoglobulins production and increase pro-inflammatory cytokine production (54). Also, ovomucoid could be used as a drug carrier since it possesses a bispecific ligand. Ovomucoid derived peptides demonstrated immunomodulating activity against T-cells; hence, they have promising potentials to be employed for pharmaceutical use in humans. All these highlight the usefulness of albumen bioactive peptides for human drugs with anticancer, antitumor and anti-hypertensive effects.

Egg white hydrolysates also protect the body against neurological and cardiovascular dysfunction induced by metals as well as preserve sperm viability by preventing membrane impairment, testis and epididymis histopathological damage (55). In rats administered 4.6 μg of Hgcl_2_ (mercury chloride) intramuscularly, the resultant effect was cardiovascular dysfunction due to high level of oxidative stress. Whereas, in rats exposed to both Hgcl_2_ and egg white hydrolysate, the egg white hydrolysate mitigated cardiovascular damage by neutralizing the activities of free radicals (56). Hence, egg white hydrolysate could be used as ingredients for functional foods that could act as a substitute for synthetic drugs to treat cardiovascular damage induced by mercury.

Egg albumen has potential in the food industry. Albumen bioactive peptides act in capacity of maintaining microbial safety of albumen as raw materials for industries and in processed foods.; ovomucoid inhibits the trypsin protease, lysozyme exerts bacteriolytic activity on food pathogens, ovomacroglobulin suppresses viral hemagglutination and ovoinhibitor inhibits bacterial and fungal serine proteases (57, 58). The bacteriolytic and antimicrobial effect of ovotransferrin on pathogenic bacteria, including *Pseudomonas* spp., *E. coli*, and *Streptococcus mutans* has been reported (59). Ovotransferrin peptide, OTAP-92, destroy Gram-negative bacteria by invading the bacterial membrane using autolysis, thereby damaging the cytoplasmic membrane (60). According to (61), ovotransferrin regulates food-poisoning activities of *E. coli* O157:H7 and *Listeria monocytogenes*. The antimicrobial capacity of lysozyme is strong compared to other albumen proteins because of its potential to regulate the proliferation of bacteria and fungi that cause food spoilage (57). *Listeria monocytogenes* and *Clostridium botulinum* which cause problems in the food industry due to its formation of toxins in plants and animals, have been successfully regulated by lysozyme activity (62). Albumen bioactive peptides could be used as natural antimicrobials to ensure food safety and reduce food spoilage.

Fresh juices, beer and wine characterized with haziness challenge consumer acceptance. To meet consumer demands in terms of clarity and sensory quality, clarifying agents are incorporated into wine during the brewing process. Albumen is used as a clarifying agent in wines to remove excess tannins and decrease wine’s astringency (55). Albumen proteins are used as raw materials in industries; egg white proteins are used as antioxidants in processed food products like cheese and sausages. Lysozyme is used as an ingredient for dentistry care products, including mouthwash, toothpaste, and chewing gums (25), because it exerts an antimicrobial effect on the oral mucosa and an inhibitory effect on periodontitis-causing bacteria. Ovomucin confers excellent texture in food products due to its protein-resistant characteristics. Hydrogels of egg white can be developed and used as bioactive material for tissue engineering applications. Owing to the health benefits of egg white bioactive peptides, they are used for health-promoting foods and drugs. It then becomes imperative to produce eggs with high albumen quality, in order to harness its usefulness as beneficial raw materials for food processing and health industry.

## Factors Affecting Albumen Quality

### Animal Health

Nutrition plays a key role in relation of laying hen’s health and product quality. Laying hens are subjected to various physiological and environmental stressors, affecting their health and productivity. In aged laying hens, lipid metabolism and fat accumulation changes are common and the fatty liver hemorrhagic syndrome is a common cause of mortality (63). Thus, regulating lipid metabolism is critical for animal health and welfare, and maintaining egg production and product quality. The lower part of the hen oviduct is open to the cloaca and subject to colonization by various microorganisms *via* the vagina; this part of the oviduct is more susceptible to infection by microbes (64), resulting in a decrease in lymphocytes and an increase in macrophages during lay. This may negatively affect laying performance and consequently egg quality. The chicken’s gastrointestinal tract is crucial to animal health; the gut microbial population and intestine morphology facilitate nutrient utilization and absorption, enhancing performance and egg quality. In late laying hens, a decline in egg production and egg quality, could be due to impaired nutrient utilization, protein metabolism, alteration of gut beneficial microbial population and low synthesis of reproductive hormones (65). Therefore, age or health status could exert detrimental influence on intestinal health and functioning capacity, leading to decline in laying performance and egg quality. In same vein, serum parameters including antioxidant enzymes; glutathione peroxidase (GSH-Px), glutathione S-transferase (GST), catalase (CAT), superoxide dismutase (SOD), total antioxidant capacity (TAOC), and oxidative biomarker: malondialdehyde (MDA) are related to oxidative stress, while immunoglobulins (IgA, IgM, and IgG) are related to immunity. The antioxidant capacity and immune functions are often used as indicators for assessing physiological status of laying hens. Nutritional strategies could be used to improve animal health *via* increased activity of antioxidant enzymes, immunoglobulin synthesis, maintenance of gut integrity which may translate into improvement in laying performance and egg quality.

According to (66), yeast can modulate the immune response and maintain a balance between innate and protective immunity in chickens by lowering 1L-1 and 1L-2. Reduced 1L-1 expression indicates a fewer number of macrophages, implying that the hens’ health is suitable for producing high-quality eggs. Recent reports showed that diet supplementation such as phytogenic feed additive (67) and natural astaxanthin (68) improved intestine morphology, small organic peptide (18) and organic selenium (69) enhanced intestinal health while alfalfa meal (70) enhanced beneficial gut microbial population. Diets such as tea polyphenols at 600 mg/kg (71) and 400 mg/kg (72), and phytogenic extracts (73) have been found to enhance immune function capacity, while magnolol enhanced the antioxidant capacity of the ovary (74). In addition, prebiotics (75, 76) improved serum antioxidant capacity, and probiotics exerted positive effect on beneficial gut microbes (77) and nutrient utilization (78). Organic trace element such as glycerol monolaurate enhanced egg quality *via* improved gut function and antioxidant capacity in late-laying hens with low health status (65). These natural products that exert positive effect on animal health and egg quality may present a mechanism of action based; on the alteration of intestinal microbiota, increased enzyme secretion, improved immune response, antioxidant activity, and morpho-histological maintenance of the GIT. Nevertheless, Miao et al. (14) reported that 800 mg/kg dietary fluoride caused damage to the liver, ovary and kidney with decreased albumen quality as resultant effect. Similarly, higher caffeine concentration in green tea decreased albumen quality and did not improve serum biochemical indices (71). These adverse effects suggest that the threshold for the inclusion level have been exceeded and animals could not tolerate this range. This is often a problem associated with synthetic feed additives.

There are several attempts to improve laying hens’ antioxidant capacity, to reduce stress and maintain high egg production. These efforts are anticipated to decrease the production of toxins likely to be transferred into the eggs and ensure safe product quality. Animal health and egg quality are inter-related because animal health influences egg quality. Thus, animal health indicators such as immune response, serum antioxidant capacity, hematology, and intestinal morphology and function may be improved with nutritional strategies, which finally culminates to enhanced egg production and quality.

### Oxidative Stress

One of the factors that negatively affect albumen quality is oxidative stress. Oxidative stress is a physiological condition in the system where the outputs of reactive oxygen species (ROS) tends to alter the vigor of antioxidant systems at either the cellular or system level (10, 79). Oxidative stress in laying hens could be due to type of diet (10, 80) and age (81–83). Oxidative stress culminate in surge of biochemical reactions including: lipid and protein peroxidation, oxidative alteration of amino acid residues, and destabilization of protein complex and function due to increased ROS (84). All these may alter the normal physiological process in the body of the animal with consequent negative effect on egg production and quality.

Increased level of oxidative products like thiobarbituric acid reactive substances and Malondialdehyde (79), methane dicarboxylic aldehyde, ketones and carbonyl protein complexes (85) both in the blood plasma and egg are linked with oxidative stress. The increased level of oxidative products often causes a reduction in activities of antioxidant enzymes (SOD, CAT, GSH-Px, and T-SOD) which are fundamental to protection of cellular structures from damaging effect of ROS (2). Adverse effects of oxidative products including; damaging of the oviduct and consequent reduction in protein synthesis (10), impacts negatively on the taste and flavor of albumen, reduce the nutritive and antioxidant capacity of egg white (86), hence poor albumen quality. Also, oxidative stress often destabilizes homeostasis of cecal microflora and destroy the immune capacity of the intestinal mucosa (77), this invariably impairs the health of the animal, nutrient utilization and consequently egg quality. Further, diet-induced oxidative stress may degrade albumen proteins and invariably albumen quality. One study reported that vanadium-based diet caused a reduction in albumen quality by decreasing the abundance of an egg white protein; lysozyme (Q6LEL2) which possesses antimicrobial properties while increasing protease inhibitors that restrain activities of some proteins (87). It is crucial to enhance endogenous antioxidant enzymes in order to mitigate the adverse effects of oxidative stress on animal and its product as well.

Endogenous antioxidant enzymes SOD and CAT that make up the antioxidant cellular enzymatic system, function as the first line of defense and are important indicators of the oxidation status of animal tissues (84). SOD catalyzes the dismutation of a superoxide anion (O_2–_) into hydrogen peroxide (H_2_0_2_) and an oxygen molecule (O_2_) to reduce the damage caused by the former. SOD also works with CAT, which converts H_2_O_2_ to H_2_O (88). This implies that enhanced antioxidant enzymes in the animal scavenge for ROS and protect the animals’ cellular structure and consequently product quality. In this line, the antioxidant capacity of egg white is enhanced, when the content and activities of antioxidant enzymes and antioxidant indicators, such as T-AOC and oxygen radical absorbance capacity “ORAC,” are increased as MDA levels are decreased (78, 86). Therefore, the use of natural antioxidants would enhance animal health and albumen synthesis and quality via: prevention of oxidative tissue damage, inhibition of oxidation and modification of nutrients.

## Nutritional Modulation of Albumen Quality

Introduction of green/safe feed additives of natural products such as prebiotics, probiotics, phytobiotics, organic trace elements and minerals, and vitamins in poultry diets could improve albumen quality. In addition, albumen quality often depends on protein secretion, which more or less is dependent on the dietary protein and amino acid intake. There is also the need to supplement in the diet of laying hens, natural antioxidants which are safer, less toxic, and have high bioavailability compared to synthetic antioxidants. These may provide eggs with albumen of high HU values, strong oxidative stability of albumen proteins, excellent rheological and technological properties, thus, better and acceptable egg quality. Adoption of nutritional strategies to enhance albumen structure with strong stability of albumen proteins, has therefore been promoted as essential means of improving albumen quality prior to lay while also improving animal health. The effects of various diets on albumen quality and animal health are presented in Supplementary Table 1.

### Probiotics and Prebiotics

Probiotics and prebiotics as natural feed additives that are non-toxic and safe for consumers, are gaining popularity as antibiotic-free alternatives in poultry nutrition. Probiotics could be used in diet of laying hens as single strains or combination of various strains (89, 90). Most commonly microorganisms used as probiotics in poultry production include colonizing species of *Streptococcus*, *Lactobacillus*, *Clostridium*, *Bacillus*, and *Enterococcus*.

Some studies have illustrated the additive effect of *C. butyricum* in improving albumen crude protein content (90) and HU value (91). In addition, probiotics such as *En. faecium* and *L. fermentum* (92), *B. subtilis* (20), *B. licheniformis* and *B. subtilis* (77), *B. velezensis* (78) and *B. amyloliquefaciens* (93) enhanced albumen quality and HU value. Similarly, albumen quality (i.e., albumen height) was improved in aged laying hens (20). Albumen nitrogen (94) and crude protein content (90) was increased in laying hens fed *Saccharomyces* fermented product and *C. butyricum*, respectively. In a likely manner like other probiotics, effective microorganisms (EM), a natural product consisting of various microbes (photosynthetic bacteria, *actinomycetes*, yeast, *Lactobacillus*) (95), enhanced albumen height and HU values (96). The improvement in albumen quality due to dietary probiotics may be associated with influence of probiotics on animal health and physiological status. Probiotics have been used in poultry nutrition to enhance activities of digestive enzymes, produce volatile bacteriostatic substances which favors breakdown of nutrient in feed, maintain gut microecological environment and improve intestinal villi morphometrics (97–99). All these facilitate nutrients absorption and utilization by the animals for albumen synthesis. For example, probiotics have been reported to enhance protein metabolism with consequent improvement in albumen quality (100). In addition, probiotics have been found to improve to enhance redox balance in animals evidenced by enhanced activities of antioxidant enzymes and secretion of immunoglobulins (93, 98). Probiotics may present improvement of antioxidant capacity, gut integrity, and immunity function as mechanisms that boost process of egg formation and albumen synthesis in laying hens. However, there are other evidences that probiotics had no significant effect on albumen quality (98, 101–104). These variations among studies may be due to probiotics constituents, dosage level and physiological status of the birds.

Prebiotics which are non-digestible oligosaccharides have been reported to exert various effects on albumen quality of laying hens. There are evidences that prebiotics such as dietary marine-derived polysaccharide (105), mannan oligosaccharides (106), yeast cell wall supplement (107) and sugar beet syrup (19), supplemented in the diets of laying hens improved albumen quality. The significant improvement in egg quality due to dietary prebiotics could be linked with beneficial effects of prebiotics on intestinal villi structures, activities of intestinal enzymes, gut environment by increasing beneficial microbes and suppression of pathogens (108, 109). These beneficial effects facilitate nutrient absorption and utilization, which are translated into improved egg production and protein synthesis. Also, prebiotics have been found to enhance ovary health, increase antioxidant and immune function (110, 111). Prebiotics influence on albumen quality may be explained by its protective effect on gut integrity, reproductive tract and oxidative stability in both serum and tissue. In contrast, Zhou et al. (112), Sozcu and Ipek (113) found no significant effect of dietary xylooligosaccharides and lignocellulose, respectively, on albumen traits. All these highlight the crucial role of diets in maintaining animal health and animal product quality, although there is less literature on influence of prebiotics on laying hens.

### Phytobiotics

The use of phytobiotics as antibiotics replacement in the poultry industry has gained much attention; consequently, its employment for enhancing performance and egg quality is expected. Phytogenics could enhance the safety and stability of eggs, which is critical for the egg-food industry. Some phytogenics supplemented in laying hen diets include some plant extracts: Chinese herbal extracts – *Lonicera confuse* and *Astragali Radix extracts* (114), ginger extract (115), and the combination of probiotics and plant extracts (116), have been reported to improved albumen quality. Other studies have also reported that phytogenics in powdery form by combination of various phytogenic feed additives (73, 117, 118), alfalfa meal (70), fruit – *Ligustrum lucidum* (119) enhanced albumen quality. However, a higher level of mulberry leaf inclusion reduced egg weight and increased HU but had no effect on albumen height (120). Furthermore, some phytogenic compounds such as Chinese herbal mixture (121), garlic and ginger root powder (122), garlic and onion powder (123), and *Ricinus communis* leaf powder at graded levels (124) had no significant effect on albumen indices and HU values. Fermented brown algae by-products, on the other hand, significantly decreased the HU value of eggs (125).

The phytogenic enhanced effect could be attributed to the beneficial effects on the oviposition process, antioxidant capacity, and efficient conversion of digested feed into eggs. The bioactive ingredients in phytogenics, such as menthol in peppermint leaves (126), the chlorogenic acid (CA) a type of phenolic acid in Chinese extracts (114), anthocyanins in grape seeds (127), and gingerols in ginger (128) have shown antimicrobial, antifungal, and antioxidant effects. This can reduce β-ovomucin degradation and invariably enhance its content in the thick albumen. In addition, the bioactive ingredients of herbal plants have been shown to improve the integrity of the magnum and uterus while aiding digestion and nutrient absorption (129). These result in increased protein synthesis, albumen secretion and ultimately higher HU values. The presence of antinutritional factors, the level of inclusion, and the nature of active ingredients may all play a role as was reported in previous works.

One economic important phytogenic additive is the green tea powder (GTP). GTP contains tea polyphenols TP, a natural antioxidant of typical flavonoids (130). The major compounds in TP are epicatechin (EC), epigallocatechin (EGC), epicatechin gallate (ECG), and epigallocatechin-3-gallate (EGCG) (131). The use of GTP as a feed additive in laying hens’ diets to improve albumen quality have been widely reported (72, 132, 133). Positive effect of tea polyphenols on albumen quality have been demonstrated in literature (134). Also, tea extracts enhanced the strength of albumen gels (135). All of which significantly enhanced albumen quality and HU value. Specifically, the improvement in albumen quality may be linked with its influence on albumen proteins. Tea polyphenols have the capacity to bind to proteins and influence their structures (136). Also, GTP supplementation increased the β-ovomucin content of thick albumen (72). In contrast, increased levels of GTP supplementation decreased albumen weight and HU value, indicating that a high dosage of caffeine may impair albumen quality (71). However, 137 reported that TP supplementation did not affect albumen indices and HU values.

The varying effects of dietary polyphenols on egg quality could be related to various factors, such as different TP components (EC, EGC, EGCG, and caffeine), TP inclusion level (71), the difference in the polyphenol compounds isolated from different plants (138), and duration of feeding. The study by Zhang et al. (5) observed significant effect of dietary EGCG on albumen quality after 8 weeks of feeding. The study of Wang et al. (80) reported that EGCG could be more effective when fed to laying birds under oxidative stress or aging. Thus, age of laying hens, tea components, physiological status and duration of feeding could account for the variations in different studies. The effect of green TP on albumen quality may be explained by some underlying proteomic mechanisms.

Proteomics reports have highlighted some mechanisms underlying the improved albumen quality of eggs from birds fed diets supplemented with green tea polyphenols. EGCG increases the level of ovalbumin (OVA)-related Y protein and decrease OVA-related X protein (32, 80). The free sulfhydryl and increased hydrophobicity properties of OVA may influence the degradation of thick albumen protein and enhance albumen height and HU values (139). TP improves albumen quality by upregulating the genes associated with cell proliferation, metal-binding mediation, and immune function-related proteins (80). Further studies are needed on the upregulation of proteins that enhance albumen quality due to dietary effects. It is imperative to investigate whether these green additives’ antioxidant and antimicrobial capacity could maintain the oxidative balance in stored eggs, thereby extending the shelf life of the eggs and ensuring the safety of consumer eggs.

### Trace Elements and Vitamins

The inclusion of organic and inorganic forms of trace elements (zinc, iron, and selenium), minerals (clay-chelates), organic acids, and vitamins in the diets of laying hens have been reported to influence albumen quality differently. Trace elements and inorganic minerals like oxides and sulfates sources (14, 140) traditionally supplemented in feeds of laying hens are presently replaced with organic microelements, especially mineral amino acid chelates and complexes due to their roles in egg quality (140). Challenges such as excretion into the environment, low bioavailability, high oxidation and destruction of nutrients (141) limits the use of the inorganic forms which also produce free radicals that could adversely affect animal health (14). Preference for the organic mineral form is promoted due to ease of absorption, high biological value, environmental friendliness, non-toxicity and safety (140). The study by Yu et al. (22) reported no significant difference between organic and inorganic zinc on egg quality. Organic forms of trace elements include selenium yeast (142), zinc-methionine (140) and iron-glycine chelate (143), which are more metabolized than inorganic forms. This suggests higher bioavailability of the organic form compared to inorganic form, such bio-fortification with microelements improves eggs’ nutritive value and biological function.

Selenium is a trace element necessary for animal and human health. Selenium have been reported to enhance reproductive performance, antioxidant and immunomodulatory function in laying hens (144, 145). In same vein, biofortification of eggs with selenium, evidenced by high content of selenium in albumen have been explored (145–149). Other evidences showed that (Se-enriched insect protein) (150) and selenium enriched yeast (148, 151), enhanced albumen height and HU. Although selenium improved albumen indices (152), no significant difference in egg quality traits was found between the Se-diet and the control group in other studies (146, 148, 153). The improvement in albumen quality may be linked with; more absorbable form of organic selenium which facilitates ease of utilization in laying hens, positive effects of selenium on oviduct health and activities of antioxidant enzymes, which may boost protein metabolism and utilization. The variations could be selenium source and dosage level. There is limited information of influence of organic selenium on albumen quality of fresh eggs.

Organic forms of zinc have been demonstrated to influence albumen quality. Qi et al. (154) reported that zinc-methionine enhanced albumen quality *via* improved T-AOC, decreased MDA level, promoted methionine synthesis, threonine and glutathione metabolism and protein metabolism. Inclusion of zinc-methionine (Zn-Met) complexes (140, 155) and zinc-methionine hydroxyl analog chelate (156) enhanced albumen HU values. (157) reported that Zn-Met improved both albumen percentage and HU. Also, Zn-Met mitigated the adverse effect of increasing age on HU values due to watery albumen (155). Besides, improving albumen quality, nutritive contents of eggs are improved *via* biofortification of eggs with increased zinc content (154, 158). The improvement in albumen quality due to dietary zinc may be accrued to the antioxidant nature of zinc and its role in metabolic process.

The liver is the primary site of zinc bioaccumulation in chickens, and it also plays a role in energy metabolism. Zinc is not an antioxidant, but it activates antioxidant enzymes and competes with redox-active transition metals such as copper and iron for binding sites. Zinc is crucial to the structure and function of Cu/Zn-SOD, accounting for about 90% of total SOD, which exerts protective effects on tissues from oxidative damage (159). In another study (160), found that dietary zinc increased metallothionein production, which inhibited lipid peroxidation, reduced MDA production, and increased Cu/Zn –SOD levels. Zinc plays an important role in protein synthesis and other biochemical reactions. (161) reported that dietary zinc regulates intestinal amino acid and protein metabolism in animals. Organic zinc has been reported to enhance total protein and globulin, serum urea nitrogen, indicating enhanced protein catabolism. Further, zinc plays a positive role in the magnum during deposition of albumen and in the isthmus during the formation of shell membrane (162). It could be deduced that the beneficial effects of zinc on intestinal integrity, protein metabolism and catabolism, antioxidant system and oviduct health may account for its positive influence on albumen quality.

Iron is one of the most critical elements for poultry and participates in various metabolic processes, including transport and storage of oxygen, protein metabolism, antioxidant and immune activity (163). Inorganic iron, FeSO_4_, is mostly used in poultry but has low absorption, pollution, and low biological value. The use of organic iron had better results due to its high bioavailability. For example, supplementation of an amino acid complexed iron (Fe-Gly) at inclusion level of 80 mg/kg in diet of laying hens, enhanced albumen quality compared to inorganic Fe (143). The result was premised on the organic iron’s higher absorption potential and easy transportation within the body, which enhances its metabolism.

In same line, organic minerals such as dietary carbo-amino-phospho-chelates in comparison to the sulphate form (inorganic) enhanced HU values (90.2 vs. 84.95) and other albumen indices in laying hens (164). Calcium in laying pigeons promoted albumen transparency (165). Dietary montmorillonite (Mineral clay) improved albumen height and HU value (166). These mineral clays can bind to pathogens and toxins in the GIT, create favorable gut microecological environment that can lead to improved performance, health status and egg quality. Glycerol monolaurate (GML), a monoglyceride of medium chain fatty acids, enhanced flavor amino acids content in the albumen (65), thereby improving the nutritive value of egg. In contrast, calcium montmorillonite (167) and amino acid-complexed manganese (168) had no significant effect on HU values and albumen quality. The variations may be due to nature of organic element or mineral used and inclusion levels in the diets.

Organic acids such as benzoic acid enhanced albumen height and HU values (169), when supplemented in diet of laying hens. In one study, supplementation of inorganic dietary fluoride at 800 and 1200 mg/kg drastically reduced albumen height and HU values, probably because of the adverse effect on the ovary and liver, leading to oxidative stress and impaired protein synthesis (14). Various studies have shown that some products extracted from plants and animals, used as feed additives influence albumen quality (74, 170, 171). Natural products including; octacosanol (171), resveratrol (170), magnolol (74), and quercetin (172) enhanced albumen quality whereas natural astaxanthin (68, 173) and antimicrobial peptide; cecropin (16) did not affect albumen quality. Age of laying hens and inclusion levels may account for inconsistencies in results among various studies. In addition, vitamins influence albumen quality; vitamin A enhanced albumen quality during heat stress *via* improved reproductive tract development (15), vitamin E (152) and 25-hydroxyvitamin D3 (174, 175), enhanced albumen HU whereas others found non-significant differences between treated groups (25-hydroxyvitamin D3) and the control (176). The improvement due to dietary vitamins could be that vitamins ensures utilization of proteins and energy by animals for health enhancement and reproduction (177).

### Amino Acid and Dietary Protein Level or Sources

The efficiency of protein utilization in diets depends on amino acid content, composition, and digestibility, hence, the deposition of albumen and yolk relies on nutrient supply (amino acids and fatty acids). Adequate essential amino acids (AA) has been traced to enhance internal and external egg quality (73), thus it is critical to supplement layers diets with AA, which cannot be synthesized in the body of laying hens. Such supplementation is critical in maintaining the structural integrity and function of the gut, decrease intestinal dysfunction and mitigate oxidative stress (178), which could lead to improved nutrient absorption and albumen synthesis. Also, egg proteins are made of AA, and the concentration of total, essential and flavor AA plays an important role in egg nutritive value and flavor. Therefore, the albumen AA profile could influence its nutritive value. Protein synthesis in the magnum may be influenced by amino acid concentration in the blood (33). Change in the level of AA supplementation in diets may alter protein synthesis resulting in a positive or negative effect on albumen quality. Liu et al. (179) reported that low methionine levels decreased the albumen ratio. In sum, amino acid plays a great role in the body’s metabolic functions, translating to an improved animal product.

Amino acid supplementation in the diets of laying hens has varying effects on albumen quality. For instance, dietary L-arginine (73), dietary L-carnitine (180), dietary threonine in the diet of old hens (181), and 0.05% purified amino acid extracted from animal blood (17) improved albumen height and HU value. Similarly, lysine supplementation enhanced albumen percentage (182). Also, dietary threonine supplemented in a dose-dependent manner increased albumen weight and percentage (183). Improved albumen indices with AA supplementation may be due to increased nutrient concentration and protein levels in the diet (184), which enhance protein synthesis (185). This, in turn, promotes the metabolic rate in the magnum, increases the activity of the shell gland, and produces eggshells with enhanced integrity, reducing the loss of carbon dioxide and improving albumen quality. In another study, threonine improved albumen percentage in a dose-dependent manner *via* upregulation of genes related to AA transport and protein deposition (183). In another study, (34), suggested that supplementing diets that enhance upregulation of AA transporters would enhance albumen synthesis and quality. On the other hand, dietary threonine (186), total sulfur amino acid (178), D-lysine (187), amino acid-complexed manganese (168) and L-citrulline, a non-protein AA (188) did not affect albumen quality. However, decreasing albumen indices with dietary AA supplementation may be due to decreased albumen synthesis (33) and the feeding regime of the AA (179). The varying results may be related to the AA inclusion level, denaturation by high temperature, and source. It has been reported that the requirements for certain essential AA for laying hens increase with an increase in the dietary level of protein (189).

Protein synthesis, which facilitates egg formation, may depend on protein utilization. The study by Kowalska et al. (190), Kuzniacka et al. (191) demonstrated that eggs of hens fed diets from different protein sources with high crude protein levels had increased albumen height, thick albumen content and HU values comparable to sources with lower crude protein level. Increased dietary crude protein (CP) level improved albumen weight, egg weight and HU value (192). Lowering CP levels from 16.49 to 14.05% (193) and from 16 to 13% (194) caused a lower HU value of 89.50 vs. 86.74 and 83.95 vs. 76.82, respectively, and invariably poor albumen quality. However, (195) reported no dietary protein levels influence on HU values. Decreased albumen indices in birds fed different dietary protein sources at different levels may be due to low dietary protein intake. (33) reported that low dietary protein intake reduces egg mass, albumen weight, and albumen solids because of the reduction in albumen secretion. Another report by (28, 196) suggests that reduction in albumen height and HU values was due to low dietary protein intake and not gossypol content of the feed. Furthermore, low albumen quality may be linked to low levels of ovomucin, a major component of albumen. The albumen ovomucin content was reduced in birds given various protein sources and levels (28). It is speculated that supplementing diets low in CP with AA could enhance albumen quality. For instance isoleucine in a low CP diet improved albumen quality (197) while threonine improved egg quality in birds fed diets with low CP levels via; enhanced secretion of digestive enzymes and increase in population of beneficial gut microflora (198). Therefore, diets from protein sources that can positively influence albumen quality *via* enhanced magnum development, increased protein synthesis, and albumen secretion should be adopted.

### Non-conventional Feedstuff

Reducing feed cost is the utmost priority of poultry farmers. Soybean meal (SBM) which is the conventional source of plant protein in most layers diet is not only expensive but has highly competitive value as food by man and other Agro-allied industries. The best strategy to decrease feed cost and competition for SBM as protein source is to formulate diets based on local alternative feedstuffs which are readily available.

Replacing SBM with 100 g/kg cotton seed meal (CSM) in layers diet enhanced albumen indices (34, 196). However, camelina or flaxseed (199), increased levels of sunflower meal (200), low gossypol cottonseed meal (LCSM), or double-zero rapeseed meal (DRM) used as the sole replacement for SBM in a layer diet (34, 35) decreased albumen height and HU values. Also, CSM at 100 g/kg reduced albumen weight, Haugh unit and albumen height (13). The reduction in albumen quality may be due to decreased tubular gland cells and epithelial growth in the oviduct magnum, slowing down protein synthesis (13). This is because synthesis of albumen proteins often occurs in the oviduct (201). However, some of the alternative feedstuffs used as a replacement for SBM such as CSM (202, 203), untreated field peas (204), wheat bran supplementation (205), full-fat flaxseed and sunflower seeds (206) had no significant effect on albumen indices and HU value. Evaluating albumen quality based on HU and albumen height may not provide enough evidence of dietary effects; investigation of albumen proteins could provide further insight into dietary effects. The study by He et al. (13) used a proteomic technique to show that albumen proteins (ovalbumin, ovotransferrin, ovomucin, lysozyme, ovoinhibitor, and clusterin) decreased in birds fed CSM at 100 g/kg. The decreased content of lysozyme and ovomucin altered the stability of the ovomucin-lysozyme complex thereby reducing albumen viscosity. Given that the albumen proteins are fundamental to albumen’s functional and technological properties, more knowledge about the protein content is needed to reach conclusions about albumen quality.

The reduction or non- significant effect on albumen indices may be due to free gossypol (FG) content of the unconventional feedstuffs. For instance, Wang et al. (34) reported that 195 g/kg low gossypol cottonseed meal impaired magnum development and function; >70 mg/kg of gossypol caused inflammatory response and damaged the mucosal immune system (203). This indicates that the gossypol level in laying hens’ diets should not exceed the above thresholds. Other evidences reported no adverse effect of FG on albumen quality and HU value (203, 207). Notably, such adverse effects could be due to the capacity of FG to inhibit several enzymes activities in the digestive tract, causing dyspepsia and growth retardation in laying birds (208). The condition also suppresses gastric secretion leading to distension of the abdomen and negative growth performance, causing clinical poisoning and liver damage (209). Alternative feedstuffs may also be low in amino acids that are crucial to protein metabolism and albumen synthesis. There are evidences that feedstuffs deficient in certain digestible AAs (arginine, phenylalanine, histidine, and leucine) (33), and indispensable amino acids (lysine, threonine, and sulfur AA) (210). Also some of alternative feedstuffs may contain antinutritional factors (i.e., phytic acid and tannin, trypsin inhibitor, vicine, and convicine) (211). Taken together, utilization of alternative feedstuffs for protein sources are limited by presence of antinutritional factors, low amino acid content and inclusion levels, which could impair nutrient absorption in the intestine, albumen secretion dimensions in the magnum and deposition of ovomucin in the albumen *in vivo*. Utilization of alternative feedstuffs may be enhanced by addition of enzymes.

Recently, the effects of these feedstuffs supplemented with exogenous enzymes have been reported (212, 213). The hypothesis developed was that the enzymes would mask the feedstuff’s antinutrients. Dietary supplementation of < 25% faba beans with enzyme (212), xylanase at 900 U/kg supplemented to corn-soybean-meal-wheat based diets (213), and 1% of non-starch polysaccharide multi-enzyme (214) enhanced albumen quality. The improvement may be due to capacity of the enzymes to unbound nutrients and make it available for utilization by animals. In contrast, exogenous xylanase at 12000 BXU/kg supplemented to non-soluble polysaccharide diets (215), xylanase at 200 mg/kg supplemented to brasetto hybrid rye diet (216), and multi-carbohydrase at 200 mg/kg supplemented to wheat-based diet (217) did not affect albumen quality. The variations may be due to feedstuff content, inclusion levels of the enzymes and type of enzyme used. Fermentation process using microorganisms may be a better strategy to degrade antinutritional factors and increase the protein content of alternative feedstuffs, thus increasing its utilization. Fermented feeds improved albumen height and HU in laying hens, probably due to favorable microbial environment which may enhance protein synthesis (218). The use of insect meal may act as a replacement of SBM as source of protein. The study of demonstrated that Black soldier larvae meal enhanced albumen weight and HU value (219). The use of readily available non-conventional feedstuffs of high biological value is most often advocated to reduce feed costs.

### Natural Oil

Inclusion of natural essential oils of plant origin in birds’ diets has been reported to influence egg quality due to their antioxidant property (220). The peppermint oil (126), peppermint oil alone or combined with thyme oil (221), fish oil (222), and essential oils (223–225), were found to improve albumen indices and HU value. The antioxidant capacity of the oils conferred by vitamin E on the plants could explain the cumulative effect of oil from diverse sources on albumen quality (226). The antioxidant compounds in the natural oil can be transferred to the egg conferring on them higher antioxidant capacity (21), that can sustain albumen quality. Natural oil from varying sources contain phenolic compounds that acts as hydrogen donors, retarding hydrogen peroxides from generating free radicals that may alter albumen synthesis. For example, oil from grape seeds improved albumen height by reducing level of oxidative products and enhancing β-ovomucin content (127). Antibacterial action of phenolic compounds such as thymol and carvacrol, may increase population of beneficial gut microbes while suppressing pathogenic bacteria (221). This would help physiological mechanisms that boost protein synthesis, by reducing competition for nutrients between the host and its microflora. In same vein, essential oils have been found to improve gut villi morphology and reproductive tract, increase secretion of digestive enzymes, leading to enhanced digestion of protein and fat and absorption in the intestine (129, 223, 227), with consequent improvement in albumen quality. It could be deduced that the improvement in albumen quality due to dietary oil, could probably be its positive effect on uterine health, oviposition process, increased pancreatic secretions, improved gut villi structures, enhanced nutrient digestion and utilization. However, camelina and flaxseed oil (199) and, soybean and linseed oil (228) reduced albumen weight but did not affect the HU values and yolk to albumen ratio. In addition, wheat bran supplemented with oil (205), combined encapsulated essential oil and organic acid (224), glycerol oil (229), essential oils (220, 227) and *Lavandula angustifolia* and/or *Mentha spicata* essential oils (194) did not exert any effect on egg weight, HU, and albumen height. The reduction in albumen quality due to natural oil supplementation may be due to anti-nutritional factors in the seeds used for oil extraction. For example, trypsin inhibitors can impair protein digestibility and albumen secretion (230). Also, high concentrations of some bioactive compounds in oils such as thymol or carvacrol may negatively influence intestinal functions and alter composition of beneficial gut microbes (231), thus harmful to the birds and invariably cause a decline in egg quality. The varying effects of oil supplementation could be due to oil sources, duration of feeding, level of supplementation and presence of antinutritional factors. Therefore, exploring oil as natural antioxidants in maintaining albumen quality of fresh and stored eggs, would be a promising research direction for improving animal product quality.

**FIGURE 4:**
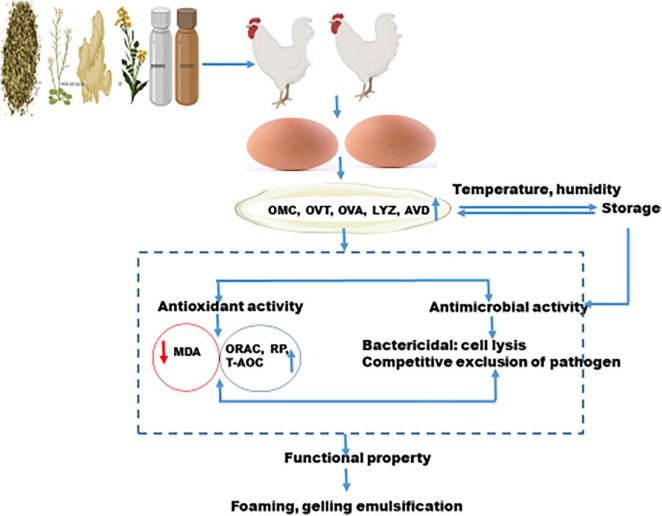
Mechanism of action of diets on albumen quality.

In summary, improving the albumen quality of laying hens fed natural feed ingredients depends on the underlying mechanism of upregulation and downregulation of oxidative products by antioxidant enzymes (Figure 4). Natural products that improve animal health would result in better albumen quality without transferring toxins to the eggs. Table 1 lists the bioactive compounds in selected natural plants and products. The bioactive compounds of these products mostly from plants, have antioxidant, antimicrobial, anti-inflammatory properties which accounts for the positive effect of natural products on albumen quality.

**TABLE 1:** Plant natural products and their bioactive compounds.

Natural products	Bioactive compounds
Red pepper	a and b -carotene
Tomatoes	lycopene
Linseed	Omega 3 and 6
Resveratrol	Natural polyphenol from grapes, peanuts
Sugar beet pulp	Phenolic acid (Ferulic)
Ginger	Gingerols, shogaol
Magnolia plant bark	Magnolol, a polyphenol
Daidzein	Flavonoid found in SB cloves
Chinese Herb mix	Flavonoids
Essential oil	Carvacrol
Natural astaxanthin	Carotenoids
Pumpkin seed meal	Lutein, a and b carotene
Walnut leaves	Ellagic acid (a phenolic compound)
Plant: Mentha amensis	Menthol, Eugenol, cineol
Plant: Geranium thunbergi	Citronellol, isomenthone
Yellow strawberry Guava leaf extract	Ellagic acid
Thyme	Carvacrol, thymol (essential oil of thyme)
Green tea	Polyphenols, catechins
Pepper mint leaves	Essential oil composed of menthol, menthone, isomenthone
Terebinth seed	Tannins
Lotus leaf extract	Catechins, quercetin
Quercetin	Flavonoids
Turmeric	Curcumin
Grape pomace	Catechins, ellagic acid
Fruit (Ligustrum lucidum)	Oleanolic acid and ursolic acid
Alquernat nebsui. L	Ellagic acid, cineol
Lonicera confuse	Chlorogenic acid
Astrgali radix	Astragalus polysaccharide
Black tea waste	Catechins
Panax ginseng Meyer leaf extract	Saponins (Ginsenosides)
Ricinus communis leaves	Phenols
Mentha spicata (spearmint) oil	Polyphenols
*Lavandula angustifolia* oil	Carvacrol, coumarin
Fennel seeds	*Trans-*anethole
Blue-green algae	Carotenoids
Almond Hulls	Polyphenols
NHDC	Natural flavonoids
Black choke berry	Procyanidins, phenolic acids
Macleaya cordata	Sanguinarine
Curcumin	Curcuminoids

## Conclusion and Future Perspectives

Albumen quality is of paramount importance to consumers, producers, breeders, and the food industry because of its biological and functional properties. In previous decades, research has focused on the performance and health of chickens, with less attention to maintaining albumen quality through nutritional modules. Various diets have been demonstrated to influence albumen quality, providing novel nutritional strategies to preserve the albumen’s integrity and the ensuing by-products. We recommend conducting similar dietary studies on albumen quality during storage to determine which diets can preserve albumen quality and reduce egg spoilage rate over time. Specifically, proteomic studies should be used to investigate the effect of diets on metabolic pathways, microbiota populations, and the proteins involved in maintaining albumen quality of fresh and stored eggs.

## Author Contributions

KQ and S-GW: conceptualization. KQ and UO: resources data, writing, and editing. UO and VO-O: writing—original draft. KQ, JW, H-JZ, and S-GW: supervision. S-GW and G-HQ: funding. All authors contributed to the reviewed and approved final version of the manuscript.

## Conflict of Interest

The authors declare that the research was conducted in the absence of any commercial or financial relationships that could be construed as a potential conflict of interest.

## Publisher’s Note

All claims expressed in this article are solely those of the authors and do not necessarily represent those of their affiliated organizations, or those of the publisher, the editors and the reviewers. Any product that may be evaluated in this article, or claim that may be made by its manufacturer, is not guaranteed or endorsed by the publisher.
