# Supplementary material for: Natural Products of Plants and Animal Origin Improve Albumen Quality of Chicken Eggs
Source: Front Nutr. 2022 Jun 10;9:875270. doi: 10.3389/fnut.2022.875270 (PMC9226613; doi:10.3389/fnut.2022.875270)
Supplement: Supplementary file 1 [file Table_1.DOC]

|  | |  | | *Table S1: Effects of Diets on Albumen Quality and Animal Health* | | | | | | | | |
| --- | --- | --- | --- | --- | --- | --- | --- | --- | --- | --- | --- | --- |
| ***Breed*** | **Age**  **(weeks)** | | **Feeding Duration (weeks)** | | **Sample Size** | **Diet Type** | **Diet and**  **Inclusion level** | **HU value Control** | **HU value Diet** | **Overall Effect on Albumen Quality** | **Overall Effect on Animal Health** | **References** |
| *Lohman Brown Lite LH* | 25 | | 8 | | 200 | PRE | 3%, 5% and 7% of sugar beet pulp (SBP) | 80.00 | 82.60 | Improved albumen quality | Reduced serum cholesterol, improved antioxidant capacity of the yolk and albumen | (1) Selim and Hussein 2020 |
| *Lohmann LSL LH* | 30 | | 20 | | 300 | PRE | 5 % and 10% sugar syrup | 90.25 | 94.86 | Enhanced albumen quality | Had no effect on liver enzymes and serum protein indices | (2) Hussein et al 2018 |
| *Single comb white leghorn* | 82 | | 25 | | 864 | PRE | 1g mannanoligosaccharides (MOS) and 24mg organic essential oil (OEO) per kg feed | 76.9 | 77.10 ns | No effect on albumen quality | Improved liver antioxidant status and increased beneficial gut microflora | (3) Bozkurt et al 2016 |
| *Lohman LSL LH* | 90 | | 9 | | 540 | PRE | 3, 6% of sugar beet pulp or wheat bran (WB), 600 mg xylanase | 80.27 | 80.53ns | No effect on albumen quality | 3% WB with enzyme improved intestine morphology and nutrient digestibility | (4) Abdollahi et al 2021 |
| *Fengda LH* | 52 | | 10 | | 3000 | PRE | 75/125 mg/kg Chitooligosaccharides (COS) | 72.69 | 74.60 ns | No effect on albumen quality | Decreased serum cholesterol, enhanced immune function and antioxidant capacity | (5) Xu et al 2020 |
| *Lohmann laying hens* | 28 | | 8 | | 1080 | PRE | Xylooligosaccharides (XOS), @ 0.1, 0.2, 0.3, 0.4 or 0.5 g/kg | 93.00 | 93.70 ns | No effect on albumen quality | Increased apparent digestibility of calcium and decreased serum cholesterol indices | (6) Li et al 2017 |
| *Hy-Line W-36 LH* | 21 | | 46 | | 256 | PRE | Yeast Cell Wall Supplement @225,450 and 900 ppm | 87.00 | 89.30 | Enhanced albumen quality | Improved performance and economic benefits | (7) Koiyama et al 2018 |
| *Hy-Line variety Brown LH* | 68 | | 8 | | 228 | PRE | IDPG (incomplete degradation products of galactomannan) @ 0.01,0.025 and 0.05% | 77.06 | 78.88 ns | No effect on albumen quality | Regulated protein metabolism and lipid metabolic profile | (8) Tao et al 2021 |
| *Hy-Line Brown LH* | 72 | | 8 | | 360 | PRO | *Enterococcus faecalis* (EF) @3.75 · 10*8 and 7.5.10*8 cfu EF/kg | 70.20 | 70.70 ns | No effect on albumen quality | Enhanced beneficial microbial population in the caecum | (9) Zhang et al 2019 |
| *Hy-Line brown LH* | 60 | | 12 | | 540 | PRO | 2:1 ratio (6.6 ×10:3.3 × 10 *B. licheniformis* yb-214245: *B. subtilis* yb-114246) | 81.80 | 89.90 | Improved albumen quality | Enhanced small intestine morphology and cercal beneficial microbial population | (10) Yang et al 2020 |
| *Hy-Line brown LH* | 49 | | 8 | | 468 | PRO | 0.1 % *B.* velezensis (*Bacillus velezensis)* | 80.46 | 89.45 | Improved albumen quality | Improved mineral nutrient retention and decreased serum cholesterol | (11) Ye et al 2020 |
| *Xufeng black bone LH* | 24 | | 12 | | 768 | PRO | 300-900 mg/kg encapsulated *Bacillus subtilis* and essential oils (BSEO) | 60.50 | 61.50 ns | No effect on albumen quality | Increased reproductive hormone levels and serum antioxidant capacity | (12) Liu et al 2020 |
| *Lohman Brown Hybrid LH* | 17 | | 21 | | 60 | PRO | 0.5 g/kg *Lactobacillus fermentum* | 79.87 | 82.51 | Improved albumen quality | NA | (13) Arpasova et al 2016 |
| *Brown ISA pullets* | 12 | | 18 | | 180 | PRO | Effective microorganism in feed and water (2ml/L, 1%) | 69.43 | 77.25 | Improved albumen quality | NA | (14) Atsbeha and Hailu 2021 |
| *Xufeng black bone chickens* | 42 | | 8 | | 432 | PRO | 250-750 mg/kg Pal-Pro complex (palygorskite and probiotics) | 64.54 | 68.32 | No effect on albumen quality | Enhanced plasma antioxidative status and immune functions | (15) Deng et al 2021 |
| *White Leghorn, Saudi black and Saudi brown* | 32 | | 13 | | 216 | PRO | 0, 200 and 400 g/t feed) probiotic (*Bacillus subtilis*) | 58.50 | 59.00 ns | No effect on albumen quality | Reduced serum cholesterol and enhanced immunoglobulins concentration | (16) Fathi et al 2018 |
| *Hy-Line brown LH* | 28 | | 24 | | 360 | PRO | *B. subtilis* CGMCC 1.921 @1.0 × 10*5 (B1), 1.0 × 10*6 (B2), 1.0 × 10^7^ (B3), and 1.0 × 10*8 (B4) cfu/g | 92.00 | 92.20 ns | No effect on albumen quality | Reduced faecal *E. coli* and enhanced beneficial gut microbiota | (17) Guo et al 2017 |
| *Hy-Line W-36 pullets* | 15 | | 34 | | 102 | PRO | *Saccharomyces* fermented product XPCTM @1.25kg/ diet | - | - | Increased albumen nitrogen | NA | (18) Martinez et al 2018 |
| *Hi-sex Brown cross LH* | day-old | | 39 | | 308 | PRO | *B. subtilis* KATMIRA 1933 (B), *B. amyloliquefaciens*  B-1895, B and C | 81.47 | 83.00 | Enhanced Haugh unit value | Improved sperm quality | (19) Mazanko et al 2018 |
| *Lohman Pink LH* | 25 | | 5 | | 8208 | PRO | 0.5g/kg *C. butyricum*, Saccharomyces | - | - | Increased crude protein content of the albumen | Decreased the oxidative products in gut (ROS) and serum (MDA) | (20) Xiang et al 2019 |
| *Shaver White Laying hens (LH)* | 19 | | 29 | | 336 | PRO | *Bacillus subtilis* (SSB: DSM29784) 2.2E+08 (medium, HSSB) CFU/kg of diet. | 65.90 | 76.60 | Improved albumen quality | Improved performance and nutrient retention | (21) Nejiat et al 2019 |
| *Jinhong -1 strain LH* | 48 | | 10 | | 960 | PRO | *C.butyricum* @2.5 x 10*4, 5 x 10*4, 1.0 x 10*5, and 2 x 10*5 cfu/g | 75.80 | 81.50 | Improved albumen quality | Enhanced immune function, antioxidant capacity and beneficial ceacal microflora | (22) Zhan et al 2019 |
| *Hy-line Brown LH* | 32 | | 16 | | 240 | PRO | *Pediococcus acidlactic* MA18/5M supplemented in and not in M and L-ED | 89.06 | 89.27 | No effect on albumen quality | Enhanced laying performance | (23) Mikulski et al 2020 |
| *Xufeng Black bone LH* | 24 | | 8 | | 480 | PRO | Probiotics (*Bacillus. Subtilis* C-3102 @ 3.0x10^5, 6 x10, 9.0 x 10 CFU/kg | 60.71 | 60.79 ns | No effect on albumen quality | Enhanced activity of GSH-Px and IgM in the serum | (24) Liu et al 2019 |
| *Lohmann Pink Shell LH* | 48 | | 9 | | 24 | PRO | *Bacillus subtilis* @0.5g/kg | 79.75 | 79.05 ns | No effect on albumen quality | Increased anti-inflammatory response and enhanced bone quality | (25) Zou et al 2022 |
| *Hy-Line Brown LH* | 28 | | 8 | | 240 | PRO | *B. amyloliquefaciens* BLCC1-238 @ 0.01, 0.03 and 0.06% | 79.50 | 85.60 | Improved albumen quality | Enhanced serum antioxidant capacity and immune function | (26) Zhou et al 2020 |
| *Lohman LSL White LH* | 46 | | 18 | | 192 | PRO | 0.3% humate (H), 0.3% probiotic (P), 0.15% humate + 0.15% probiotic (HP) | 79.65 | 79.34 ns | No effect on albumen quality | Increased MUFA in the yolk and yolk colour | (27) Macit et al 2021 |
| *Hy-Line Brown LH* | 40 | | 5 | | 192 | PRO | 0.5 g kg-1 *Bacillus subtilis* RX7, 0.5 g kg-1 *Bacillus subtilis* B2A 1.0x10*9 | 94.51 | 95.55 ns | No effect on albumen quality | Decreased pathogenic bacteria in the excreta | (28) Shi et al 2020 |
| *Hy-Line Brown LH* | 26 | | 12 | | 180 | PRO | Microencapsulated *E. faecalis* 100 mg / kg | 74.76 | 77.00 | Improved Haugh unit | Enhanced serum biochemical indices and diversity of caecal microflora | (29) Song et al 2019 |
| *White Plymouth Rock and Red Rhodes Island LH* | 50 | | 16 | | 64 | PRO | *Bacillus subtilis* PB6 @500g/t, chromium propionate (CrProp) 50g/t, and PB6 +(CrProp) | 88.70 | 90.60 ns | No effect on albumen quality | Improved productive performance and egg shell quality | (30) Souza et al 2021 |
| *Hy-Line Brown Pullets* | 27 | | 12 | | 384 | PRO | *B. subtilis*/ Galli Pro Max, *B. Licheniformis*/ Galli Pro Tect @ 8 x10*5 CFU/kg | 89.42 | 91.02 ns | Improved albumen height | Reduced population of pathogenic bacteria in the caecum | (31) Upadhya et al 2019 |
| *White Leghorn LH* | 42 | | 12 | | 540 | PRO | Herbal extract (Phytogrow), probiotic product and coated butyric acid @500g/ton | - | - | No effect on albumen quality | Phytogrow reduced count of pathogenic bacteria | (32) Bhagwat Vishwanath et al 2020 |
| *Hy-Line Brown LH* | 59 | | 8 | | 384 | PRO | *C.butyricum* @ 0.3 g/kg (2.8 x10*8),0.9 g/kg (8.4x10*8), 2.7 g/kg (2.5x10*9) | 78.46 | 80.55 ns | No effect on albumen quality | Improved feed efficiency and intestinal absorption function | (33) Wang et al 2020 |
| *Charoen Pokphand (CP) brown hens* | 25 | | 4 | | 108 | PRO | Probiotics + dried yeast | 93.60 | 95.40 | Enhanced albumen quality | Enhanced yolk colour and reduced expression of inflammatory cytokines | (34) Khochamit et al 2021 |
| *Hy-Line Brown LH* | 67 | | 8 | | 60 | PRO | Yeast culture @ 3.0g/kg diet | 92.10 | 94.00 ns | No effect on albumen quality | Upregulated digestive enzyme activities and intestinal health-related genes | (35) Zhang et al 2020 |
| *Broiler breeder hens ROSS 308* | 57 | | 7 | | 20000 | PRO | *Bacillus subtilis* PB6 (2 × 10^7^ cfu/g) at the rate of 1 g/kg of diet | 88.00 | 92.00 | Improved albumen quality | Enhanced production, animal health and economic returns | (36) Darsi and Zhaghari 2021 |
| *Hy-Line Brown LH* | 34 | | 8 | | 288 | AA | 0.25%, 0.50% and 1.00% L-Citrulline | 73.91 | 75.53 ns | No effect on albumen quality | Increased serum antioxidant capacity and reduced lipid peroxidation | (37) Uyanga et al 2020 |
| *Hi-sex Brown LH* | 33 | | 16 | | 504 | AA | Dietary digestible arginine levels: 880, 968, 1056, or 1144 mg/kg o | - | - | Increased albumen percentage | 1144 mg/ kg enhanced immune status | (38) Fascina et al 2017 |
| *Hy-Line Brown LH* | 20 | | 28 | | 960 | AA | Threonine (6.14, 6.98, 8.75, 10.06 and 10.55 g/kg) and chelated Zn (31, 73, 104 and 121 mg/kg | - | - | No effect on albumen quality | Higher Zn levels reduced nutrient efficiency and energy intake. | (39) Neto et al 2020 |
| *Lingnan Yellow Feathered LH* | 49 | | 18 | | 720 | AA | Threonine @ 0.38% (Basal diet) and 0.12, 0.24,0.36,0.48 or 0.60% supplemented in diets | 74.70 | 77.80 ns | No effect on albumen quality | Enhanced upregulation of genes related to amino acid transportation and protein deposition | (40) Jiang et al 2019 |
| *Hy-Line W-36 LH* | 19 | | 26 | | 144 | AA | L-methionine added to obtain :70%, 85% and 100% of TSAA | - | - | No effect on albumen quality | TSAA levels improved bone metabolism in the animals | (41) Castro et al 2020 |
| *Shaver white pullets* | 19 | | 27 | | 179 | AA | isoleucine @ 70,80,90 and 100 % to low CP and AA diet | 68.33 | 71.59 | Enhanced albumen quality | Improved performance with isoleucine supplementation | (42) Parenteau et al 2021 |
| *Commercial LH* | 56 | | 8 | | 144 | AA | 0.05% Purified Amino Acid form animal blood | 72.53 | 81.28 | Improved albumen quality | Reduced inflammatory cytokines and blood chemical variable were normal | (43) Joshi et al 2019 |
| *Fengda No.1 laying hens* | 33 | | 8 | | 960 | AA | Dietary valine @ 0.59, 0.64, 0.69, 0.74, and 0.79% | 74.45 | 69.40 | increasing levels reduced albumen quality | Enhanced amino acid nutrient uptake and utilization | (44) Jian et al 2021 |
| *Hy-Line Brown LH* | 84 | | 4 | | 340 | ENZY | LE (low energy diet) + 0.04% or 0.08% β-mannanase | 80.90 | 83.40 | No effect on albumen quality | No significant effect on nutrient utilization | (45) Kim et al 2017 |
| *Hy-Line Brown LH* | 44 | | 12 | | 240 | ENZY | Xylanase @900 U/kg supplemented to Corn-Soybean-meal-Wheat based diets | 89.92 | 93.22 | Improved albumen quality | Increased excreta lactic acid bacteria | (46) Lei et al 2018 |
| *Hy-Line Brown LH* | 25 | | 8 | | 144 | ENZY | High NSP level (at 13.3 or 10.8 g/kg) and exogenous Xylanase @ 12,000 BXU/kg | 99.40 | 100.80 ns | No effect on albumen indices | Xylanase induced higher laying performance | (47) Nguyen et al 2021 |
| *ISA Brown LH* | 17 | | 8 | | 240 | ENZY | Brasetto hybrid rye @0.10.15,20. or 25% and with or without Xylanase @200mg/g feed | 90.20 | 89.70 ns | No effect on albumen quality | Reduced intestinal viscosity but did not influence egg production positively | (48) Lojewska et al 2019 |
| *Hy-Line Brown LH* | 18 | | 10 | | 432 | ENZY | 0.1% of NSP multi enzyme | 91.24 | 92.74 ns | No effect on albumen quality | Increased nitrogen digestibility and decreased excreta ammonia excretion | (49) Sun and Kim 2019 |
| *Hy-Line W-36 LH* | 62 | | 10 | | 120 | ENZY | Rice bran (0, 15, and 25%) and with or without phytase supplementation (250 FTU/kg). | 72.43 | 73.41 ns | No effect on albumen quality | Reduced excretion of phosphorus and Calcium to the environment | (50) Habibollahi et al 2019 |
| *Bovans White LH* | 50 | | 12 | | 384 | ENZY | Multicarbohydrase (MC) @0,200 mg/kg and butyrate glycerides (BG) 0,2 g/kg to a wheat-based diet | 71.21 | 70.68 ns | No effect on albumen quality | Enhanced intestinal health and beneficial gut microbial population | (51) Arabshahi et al 2021 |
| *Hy-Line Brown LH* | 20 | | 8 | | 162 | NCF | Cottonseed meal @ 6 or 12% | 92.50 | 91.30 ns | No effect on albumen quality | Reduced laying performance | (52) Mu et al 2019 |
| *Hy-Line W36 LH* | 23 | | 8 | | 100 | NCF | 7.5 and 15% of prime hulls;7.5 and 15% of California type hulls | 105.80 | 106.80 ns | No effect on albumen quality | Enhanced nutrient digestibility and no adverse effect on egg production | (53) Wang et al 2021 |
| *Hisex Brown LH* | 20 | | 22 | | 160 | NCF | 100% Faba Beans &250mg enzyme | 65.34 | 97.26 | Improved albumen quality. | NA | (1) Abdel -Hack et al 2015 |
| *Babcock Brown LH* | 27 | | 8 | | 150 | NCF | 13.5% full-fat whole flaxseed seeds and 13% full-fat ground sunflower seeds | 92.40 | 92.50 ns | No effect on albumen quality | Enhanced PUFA content of eggs but decreased production | (54) Agullion -Paez et al 2020 |
| *White Leghorn Layers* | 62 | | 8 | | 72 | NCF | Broccoli Floret residue @0,40,80 and 120 g/kg | 82.00 | 82.80 ns | No effect on albumen quality | Reduced total tract digestibility coefficients and nitrogen retention | (55) Mustafa and Baurhoo 2018 |
|  |  | |  | |  | NCF | CSM @19.5, 67.5, 115.5, and 163.5 g/kg CSM, in which free gossypol (FG) level was 20, 70, 120, 170 mg/kg respectively | 79.10 | 79.79 ns | No effect on albumen quality | FG beyond 70 mg /kg reduced immunity of ileum mucosa and caused liver damage | (56) Wang et al 2020 |
| *Lohmann Pink-shell LH* | 31 | | 12 | | 1080 | O | Rapeseed oil @2 and 4 % levels | 70.22 | 73.55 | The sources Improved albumen quality | Reduced serum triglycerides | (57) Yuan et al 2019 |
| *ISA Brown LH* | 27 | | 50 | | 4000 | O | Essential oil (Eugenol @300 mg/kg diet | 79.72 | 85.32 | Improved albumen quality | Improved immunity functions of the birds | (58) Ghanima et al 2020 |
| *Lohmann LH* | 54 | | 12 | | 960 | O | Essential oil (Eniva O) @0,50.100.150 mg/kg diet | 84.14 | 82.95 ns | No effect on albumen quality | NA | (59) Ding et al 2017 |
| *Hy-Line Brown LH* | 28 | | 19 | | 216 | O | 8 % fish oil | - | - | Enhanced albumen quality | Increased oxidative indices in the serum | (60) Dong et al 2018 |
| *Hy-Line W36 LH* | 40 | | 10 | | 150 | O | Herbal essential oil @100,200 mg/kg | 83.35 | 86.89 | Improved albumen quality | Improved hepatic antioxidant response and reduced serum lipid profile | (61) Mousavi et al 2017 |
| *Hysex Brown LH* | 49 | | 6 | | 144 | O | Crude palm oil @ 20,30, 40 g/kg diet | 79.80 | 80.80 ns | No effect on albumen quality | Increased concentration of vitamin E in the animal adipose tissue | (62) Areeob et al 2018 |
| *Lohmann Brown hens* | 23 | | 16 | | 144 | OE | 0.3 mg/kg of organic Se from *Stenotrophomonas maltophilia* (bacterial organic Se, ADS18). | 52.39 | 70.86 | Improved albumen quality | Enhanced intestine morphology and beneficial caecum microbes | (63) Muhammad et al 2021 |
| *Lohmann Pink hens* | 29 | | 10 | | 360 | OE | 0.5g/kg MMT (montmorillonite). Aluminosilicate Mineral clay | 72.90 | 79.00 | Enhanced albumen quality | Improved antioxidant and immune status of the animal | (64) Chen et al 2019 |
| *Lohmann Brown LH* | 38 | | 22 | | 135 | OE | 0,0.5 and 1% Sepiolite | 82.10 | 81.76 | No effect on albumen quality | Reduced serum cholesterol and triglyceride | (65) Yalcin et al 2016 |
| *Roman LH* | 21 | | 6 | | 720 | OE | Organic (zinc amino acid complex) and inorganic (ZnSO4) @, low (35 mg/kg) and high (70 mg/kg) | 60.30 | 61.78 ns | No effect on albumen quality | Improved antioxidant capacity by increasing serum Cu-Zn SOD and SOD | (66) Yu et al 2020 |
| *Hy-Line Brown LH* | 40 | | 24 | | 252 | OE | 300 mg/kg of glycerol monolaurate (medium-chain a-monoglycerides) | 90.06 | 94.35 | Enhanced flavour amino acid content of albumen | Enhanced reproductive hormones level and Improved gut beneficial microbes | (67) Liu et al 2020 |
| *Chinese Yellow Broiler Breeder Hens* | 30 | | 15 | | 576 | OE | Dietary Copper @13.5 mg /kg diet | - | 79.5 | Enhanced albumen quality | Beyond 13 mg/kg induced oxidative stress | (68) Gou et al 2020 |
| *Hy-Line Brown LH* | 30 | | 12 | | 1152 | OE | 0.3 mg/kg Se from sodium selenite and 0.1, 0.2, 0.3, or 0.4 mg/kg of Se from SY (selenium enriched yeast) | 77.12 | 77.18 ns | No effect on albumen quality | SY enhanced immune and antioxidant status of the animal | (69) Lu et al 2020 |
| *Roman LH* | 21 | | 8 | | 720 | OE | 0.3 /0.5 mg selenium yeast and 0.3/0.5 sodium selenium (Control) | 75.01 | 75.62 ns | No effect on albumen quality | No effect on blood biochemical indices (albumin, total protein and urea nitrogen | (70) Liu et al 2020 |
| *Jing-Hong LH* | 50 | | 11 | | 288 | OE | 0 mg/kg S e from sodium selenite (SS), 0.3 mg Se /kg from Selenium yeast (SY) and SS + SY (0.15 +0.15) | 73.50 | 74.70 ns | No effect on albumen quality | Enhanced antioxidant capacity of the laying hens | (71) Han et al 2017 |
| *Hyline Variety White LH* | 26 | | 8 | | 810 | OE | 80 mg Fe/kg from Fe-Glycine | 77.02 | 87.05 | Enhanced albumen quality | Fe-Glycine enhanced iron transport and metabolism in the animals | (72) Xie et al 2019 |
| *Hy-Line Gray LH* | 37 | | 7 | | 384 | OE | Dietary fluoride @ 400, 800 and 1200 mg /kg | 85.09 | 83.03 | 800 and 1200 mg reduced albumen height | Damaged tissues; ovary, liver and kidney | (73) Miao et al 2019 |
| *Jinghong-1 strain LH* | 49 | | 10 | | 720 | OE | Zn-Meth (Zinc methionine @ 80 and 100 mg /kg | - | ** | Enhanced albumen quality | Improved hepatic and antioxidant status of the laying hens | (74) Li et al 2019 |
| *Hisex Brown LH* | 22 | | 12 | | 120 | OE | 100 mg/kg Zinc -Methionine | 82.26 | 88.67 | Enhanced albumen quality | Increased Cu-Zn-SOD serum activity and decreased serum triglyceride | (75) Abdel -Hack et al 2018 |
| *Hy-Line Brown LH* | 21 | | 24 | | 756 | OE | Amino -acid complexed-manganese (Mn) @ 20,40,80,120,400 or 800 mg /kg | 76.73 | 77.78 ns | No effect on albumen quality | 80 mg/kg enhanced serum antioxidant capacity | (76) Cui et al 2019 |
| *Delkab White LH* | 53 | | 24 | | 320 | OE | Carbon-amino-phospho-chelates (Fe, Cu, Mn, Se, Zn) @43.7 mg/kg | 84.95 | 90.20 | Enhanced albumen quality | NA | (2) Pereira et al 2018 |
| *Hy-Line Brown LH* | 64 | | 10 | | 270 | GT | 200 mg/kg tea polyphenols (TP200) | 72.94 | 81.60 | Enhanced albumen quality | Enhanced intestine integrity and Magnum morphology | (77) Wang et al 2018 |
| *Huainan partridge hens* | 20 | | 12 | | 460 | GT | 1% green Tea powder (GTP) | 67.50 | 73.65 | Enhanced albumen quality | NA | (78) Zhang et al 2020 |
| *Lohmann commercial LH* | 63 | | 8 | | 288 | GT | 600 mg /kg Tea Polyphenols | 94.00 | 96.76 | Enhanced albumen quality | Improved antioxidant status in the liver, ovary and serum | (79) Zhou et al 2021 |
| *Lohmann LH* | 35 | | 8 | | 288 | GT | 165 mg/kg EGCG | 81.82 | 88.13 | Enhanced albumen quality | Enhanced serum antioxidant capacity | (80) Wang et al 2020 |
| *Lohmann LH* | 35 | | 8 | | 720 | GT | 400 mg /kg Tea polyphenols | 83.82 | 87.13 | Enhanced albumen quality | Upregulation of antioxidant related proteins | (81) Ding et al 2020 |
| *Lohmann Pink-Shell LH* | 36 | | 12 | | 840 | GT | Caffeine concentration @ 37.5 mg/kg, | 93.5 | 89.7 | Higher concentration decreased albumen quality | No effect on serum antioxidant status | (82) Zhu et al 2020 |
| *Xianju Chickens* | 20 | | 10 | | 240 | GT | 3% green tea powder | 74.82 | 82.07 | Enhanced albumen quality | NA | (83) Xia et al 2018 |
| *Lohmann LH* | 24 | | 12 | | 180 | GT | 6% Black tea waste | 83.47 | 88.32 | Enhanced albumen quality | Decreased serum cholesterol | (84) Kaya et al 2018 |
| *HI-Sex Brown LH* | 33 | | 16 | | 504 | PHY | Phytogenic additive *(Baccharis dracunculifolia* (40%), *Astragalus membranaceus* lipopolysaccharides (20%), cinnamon, and grape seed (20%). | 86.48 | 88.46 | Enhanced albumen quality | Improved immune and health status of the birds | (38) Fascina et al 2017 |
| *Hy-Line Brown LH* | 40 | | 8 | | 288 | PHY | Ginger extract @100g/ton. | 76.88 | 85.37 | Enhanced albumen height | Enhanced antioxidant status of the animal | (85) Wen et al 2019 |
| *ISA-Brown LH* | 16 | | 8 | | 216 | PHY | Basal diets, extracts of ginger (0.0032%) and, ginger (0.0016%) and thyme (0.0016%) combined. | 83.5 | 86.7 | Enhanced albumen quality | NA | (86) Damaziak et al 2018 |
| *Bovans Brown commercial LH* | 30 | | 12 | | 180 | PHY | 0.5% garlic powder (GP), 1% GP, 1% onion powder (OP), 1% OP + 0.5% GP, and 1% OP + 1% GP} | 86.80 | 86.40 ns | No effect on albumen quality | Decreased serum cholesterol | (87) Omer et al 2019 |
| *Bovans LH* | 80 | | 12 | | 96 | PHY | Grape pomace @ 4 or 6% | 82.71 | 85.87 | No effect on albumen quality | Reduced serum glucose level but no effect on serum cholesterol nor triglycerides | (88) Kara et al 2016 |
| *Tetra SL- 1.1 LH* | 50 | | 6 | | 120 | PHY | 9% rapeseed meal and 3% grapeseed meal, T2 control diet with 9% flaxseed meal and 3% sea buckthorn meal, S | 82.12 | 85.97 | Enhanced Haugh unit value | Improved health related lipid indices | (89) Vlaicu et al 2021 |
| *Hy-Line Brown LH* | 30 | | 3 | | 60 | PHY | 30, 50 mg Curcumin /kg diet | 81.26 | 85.41 | No significant effect on albumen quality | Exerted anticoccidial effect on the laying hens | (90) Galli et al 2018 |
| *Lohmann Brown LH* | 30 | | 6 | | 40 | PHY | Basal diet and basal diet + 0.5% sumac or 0.5% turmeric or 0.25% sumac + 0.25% turmeric | 100.2 | 99.6 ns | No effect on albumen quality | No effect on serum biochemical indices | (91) Gumus et al 2018 |
| *Hy-Line W-36 LH* | 60 | | 9 | | 90 | PHY | 0.5ml/L phytogenic compound (*Alquetnat nebusi. L*) | 88.97 | 93.19 | Enhanced albumen quality | Enhanced the villi width of the duodenum | (92) Sharma et al 2020 |
| *Hy-Line Brown LH* | 40 | | 32 | | 156 | PHY | *Salvia officinalis L*. at 0.5% or 1.0%, | 91.77 | 92.17 ns | No effect on albumen quality | NA | (93) Galamatis et al 2021 |
| *ISA Brown LH* | 45 | | 4 | | 75 | PHY | Strawberry guava leaf extract @0.0, 0.05, 0.10, 0.15, and 0.20/kg feed) | 97.40 | 95.90 ns | No effect of albumen quality of fresh eggs | Reduced serum lipid peroxidation and enhanced antioxidant status | (94) Dos Santos et al 2020 |
| *ISA -Brown LH* | 28 | | 16 | | 96 | PHY | *Mentha arvensis* (MA) and *Geranium thunbergii* (GT) extracts   (0.1% 1 MA:1 GT) in drinking water | 70.07 | 76.96 | Enhanced albumen quality | Enhanced the serum immunity of the animal | (95) Dilawar et al 2021 |
| *Beijing -you Chickens* | 20 | | 8 | | 600 | PHY | Alfalfa meal, @ 10% | 83.39 | 89.00 | Enhanced albumen quality | Stimulated proliferation of gut beneficial bacteria | (96) Zheng et al 2019 |
| *Tetra SL-lh* | 50 | | 8 | | 80 | PHY | Pumpkin seed meal @9% | 89.73 | 92.22 ns | No effect on albumen quality | NA | (97) Vlaicu and Panaite et al 2020 |
| *Hy-Line commercial Hybrid LH* | 42 | | 20 | | 480 | PHY | Dried fruit pomace of blackchoke berry and black currant @30g/kg diet | 80.65 | 82.00 ns | No effect on albumen quality | Improved the immunological status of the laying hens | (98) Czajka and Skomorucha 2021 |
| *Xuefeng black-bonechickens* | 47 | | 12 | | 576 | PHY | *Macleaya cordata* extract (MCE) @100 ,150 or 200 mg/kg diet | 62.21 | 60.11 ns | No effect on albumen quality | Enhanced reproductive hormones levels and immune activity | (99) Guo et al 2021 |
| *Bankiva line LH* | 40 | | 12 | | 600 | PHY | 0-1% marine microalga (*Dunaliella salina*) | 91.30 | 90.19 ns | No effect on albumen quality | Improved performance and egg oxidative stability | (100) Fernandes et al 2020 |
| *Norfa Laying hens* | 38 | | 8 | | 160 | PHY | 0-0.3% *Spirulina platensis* (Blue-green algae) | 83.60 | 87.90 | No effect on albumen quality | Decreased serum cholesterol and increased hepatoprotective activity | (101) Selim et al 2018 |
| *Hy-Line Gray LH* | 50 | | 4 | | 240 | PHY | *Lactobacillus plantarum* (L. plantarum) and fermented Astragalus @ 3% | 92.57 | 93.80 ns | No effect on albumen quality | Enhanced antioxidant, immune function and ileal microbiota | (102) Shi et al 2020 |
| *Hy-Line Brown LH* | 48 | | 6 | | 135 | PHY | 0.3 % SPA (fermented *S. chinensis* pomace, fermented pine needle extract, and Chinese chive powder in the ratio of 2:2:1. | 88.65 | 92.20 | Enhanced albumen quality | Increased serum phosphate content while no effect on immunity status was observed | (103) Moon et al 2021 |
| *Hy-Line W-36 Leghorn Layers* | 36 | | 8 | | 200 | PHY | Sargassum (*Sargassum hemiphyllum* var.) @ 1-5% dietary inclusion | 63.33 | 66.02 ns | No effect on albumen quality | Enhanced immunity status | (104) Fan et al 2021 |
| *Jinfeng Chickens* | 79 | | 18 | | 540 | PHY | 2% and 4% of Fresh lemon | 77.00 | 79.80 | Enhanced albumen quality | Improved serum antioxidant capacity, immune function and lipid metabolism | (105) Wan et al 2021 |
| *Lohmann Brown LH* | 72 | | 6 | | 360 | PHY | Fruit (*Ligustrum lucidum* @0.25 % in powder form | 72.33 | 76.52 | Enhanced albumen quality | Reduced serum triglyceride and increased high-density-lipoprotein cholesterol | (106) Li et al 2017 |
| *Hy-Line Brown LH* | 72 | | 8 | | 360 | PHY | *Ligustrum lucidum* (Chinese fruit) in powder @1 and 2% | 70.24 | 70.86 ns | No effect on albumen quality | Enhanced beneficial caecum microbial population | (107) Chen et al 2020 |
| *Lohmann-Pink-Shell LH* | 52 | | 12 | | 1440 | PHY | 0.2% *Lonicera confusa* and *Astragali radix* extracts 1:1 | 68.34 | 71.80 | Enhanced albumen quality | Enhanced antioxidant status and reduced systemic inflammation | (108) Xie et al 2019 |
| *Beijing PINK-1 LH* | 60 | | 8 | | 360 | EP | Resveratrol @ 0.5, 1.0, 2.0 and 4.0 g/kg | 68.7 | 73.1 | Enhanced albumen quality | Enhanced serum antioxidant status and reduced cholesterol levels | (109) Feng et al 2017 |
| *Lohmann-Pink-Shell LH* | 45 | | 16 | | 720 | EP | 200mg/kg benzoic acid | 82.6 | 86.19 | Enhanced albumen quality | Enhanced intestinal morphology and beneficial gut microbial population | (110) Gong et al 2021 |
| *Hy-Line Brown LH* | 20 | | 12 | | 480 | EP | *Haematococcus pluvialis* powder to provide 0, 21.3, 42.6, and 213.4 mg of astaxanthin per kilogram of feed | 90.41 | 89.01 ns | No effect on albumen quality | Improved serum and liver antioxidant status but inclusion up to 213.4 mg/kg should be avoided | (111) Dansou et al 2021 |
| *Nogenda No 3 LH* | 50 | | 4 | | 450 | EP | Natural Astaxanthin from (Microalga; *Haematococus pluvalis*) @0,20,40,80 or 160 mg /kg | 83.40 | 83.34 ns | No effect on albumen quality | NA | (112) Heng et al 2020 |
| *JingfenPink-shell LH* | 50 | | 12 | | 360 | EP | Magnolol (Plant polyphenol) @100 mg/kg | 68.44 | 74.66 | Improved albumen quality | Enhanced hepatic lipid metabolism, antioxidant capacity of ovary and intestinal mucosa integrity | (113) Chen et al 2021 |
| *Lohmann Commercial LH* | 66 | | 10 | | 240 | EP | 400 mg NHDC /kg diet (neohesperidin dihydrochalcone) | 80.11 | 86.54 | Increased albumen indices and albumen gel properties | Improved serum biochemistry and Intestinal morphology | (114) Zhu et al 2021 |
| *Hy-Line Brown LH* | 60 | | 8 | | 360 | EP | Daidzein @ 0.03kg /t and Chinese Herb @0.6kg/t | 76.00 | 79.20 | Improved albumen quality | enhanced levels of reproductive hormones | (115) Zhang et al 2021 |
| *Hy-Line Brown LH* | 72 | | 8 | | 360 | EP | Antimicrobial peptide (AMP) @ 50 mg or 100 mg/kg: Cecropin A (insect) | 70.24 | 70.44 ns | No effect on albumen quality | Exerted beneficial effects on caecum microbiota | (116) Chen et al 2020 |
| *Hy-Line Brown LH* | 56 | | 12 | | 432 | EP | Upro a peptide @ 0.8%) to replace SBM | 75.45 | 85.45 | Enhanced albumen indices and textural quality | Enhanced intestinal health and morphology | (117) Chang et al 2021 |
| *Lohmann white LH* | 28 | | 8 | | 96 | EP | Flavonoids: quercetin @0.5g/kg | 79.64 | 75.73 | Decreased albumen indices | NA | (118) Iskender et al 2017 |
| *Lohmann LH* | 18 | | 35 | | 100 | Insect meal | Insect larvae meal (fermented sago larva) replacing fish meal@ 0, 15, 25, and 40% | 66.50 | 66.60 ns | No effect on albumen quality | NA | (119) Adli 2021 |
| *Single Comb White Leghorn hens* | 43 | | 4 | | 216 | Insect meal | Black Solider Fly larvae meal @ 24% | 90.83 | 90.72 ns | No effect on albumen quality | NA | (120) Patterson et al 2021 |
| *Bovans Brown LH* | 42 | | 12 | | 135 | VIT | Vitamin A @ 8000 and 16000 IU/kg diet | 77.00 | 79.00 | Enhanced albumen quality | Improved haematology indices and reduced effect of heat stress | (121) Abdel -Hack et al 2019 |
| *Lohmann Brown hens* | 45 | | 16 | | 800 | VIT | Dietary 25-OH-D3 levels (0 and 69 mg/kg) | - | - | No effect on albumen quality | Improved the skeletal health of the animal | (122) Wang et al 2020 |
| *Hy-Line W36 LH* | day old | | 95 | | 392 | VIT | D3 at 2,760 IU/kg (D); D3 at 5,520 IU/kg (DD), and D3 at 2,760 IU/kg plus 25OHD at 2,760 IU (69 mg)/kg (25D). | 90.00 | 95.70 | Enhanced albumen quality not beyond week 33 | NA | (123) Chen et al 2020 |

LH- laying hens, PRO-probiotics, PRE-Prebiotics, PHY-phytobiotics, GTP-Green tea, NCF-non-conventional feedstuffs, ENZY-enzymes, OE- Organic race elements, O-Oil AA-amino acids, EP-extracted product, VIT-Vitamins. NA- not evaluated, ns- not significant, -- (control diet not available)

1. Selim S, Hussein E. Production performance, egg quality, blood biochemical constituents, egg yolk lipid profile and lipid peroxidation of laying hens fed sugar beet pulp. Food Chemistry. 2020;310:125864.

2. Hussein AS, Ayoub MA, Elhwetiy AY, Ghurair JA, Sulaiman M, Habib HM. Effect of dietary inclusion of sugar syrup on production performance, egg quality and blood biochemical parameters in laying hens. Animal Nutrition. 2018;4(1):59-64.

3. Bozkurt M, Bintas E, Kirkan S, Aksit H, Kucukyilmaz K, Erbas G, et al. Comparative evaluation of dietary supplementation with mannan oligosaccharide and oregano essential oil in forced molted and fully fed laying hens between 82 and 106 weeks of age. Poult Sci. 2016;95(11):2576-91.

4. Abdollahi A, Karimi A, Sadeghi A, Bedford M, Ashengroph M. The effects of the fiber source and xylanase supplementation on production, egg quality, digestibility, and intestinal morphology in the aged laying hen. Poultry Science. 2021;100(3):100936.

5. Xu Q, Azzam MMM, Zou X, Dong X. Effects of chitooligosaccharide supplementation on laying performance, egg quality, blood biochemistry, antioxidant capacity and immunity of laying hens during the late laying period. Italian Journal of Animal Science. 2020;19(1):1180-7.

6. Li D, Ding X, Zhang K, Bai S, Wang J, Zeng Q, et al. Effects of dietary xylooligosaccharides on the performance, egg quality, nutrient digestibility and plasma parameters of laying hens. Animal Feed Science and Technology. 2017;225:20-6.

7. Koiyama N, Utimi N, Santos B, Bonato M, Barbalho R, Gameiro A, et al. Effect of yeast cell wall supplementation in laying hen feed on economic viability, egg production, and egg quality. Journal of Applied Poultry Research. 2018;27(1):116-23.

8. Tao Y, Wang T, Huang C, Lai C, Ling Z, Zhou Y, et al. Production performance, egg quality, plasma biochemical constituents and lipid metabolites of aged laying hens supplemented with incomplete degradation products of galactomannan. Poultry Science. 2021;100(8):101296.

9. Zhang Y, Ma W, Zhang Z, Liu F, Wang J, Yin Y, et al. Effects of Enterococcus faecalis on egg production, egg quality and caecal microbiota of hens during the late laying period. Archives of animal nutrition. 2019;73(3):208-21.

10. Yang J, Zhan K, Zhang M. Effects of the use of a combination of two Bacillus species on performance, egg quality, small intestinal mucosal morphology, and cecal microbiota profile in aging laying hens. Probiotics and antimicrobial proteins. 2020;12(1):204-13.

11. Ye M, Wei C, Khalid A, Hu Q, Yang R, Dai B, et al. Effect of Bacillus velezensis to substitute in-feed antibiotics on the production, blood biochemistry and egg quality indices of laying hens. BMC Veterinary Research. 2020;16(1):1-8.

12. Liu X, Liu W, Deng Y, He C, Xiao B, Guo S, et al. Use of encapsulated Bacillus subtilis and essential oils to improve antioxidant and immune status of blood and production and hatching performance of laying hens. Italian Journal of Animal Science. 2020;19(1):1573-81.

13. Arpášová H, Kačániová M, Pistová V, Gálik B, Fik M, Hleba LJSPAS, et al. Effect of Probiotics and Humic Acid on Egg Production and Quality Parameters of Laying Hens Eggs. 2016;49(2).

14. Atsbeha AT, Hailu TG. The Impact of Effective Microorganisms (EM) on Egg Quality and Laying Performance of Chickens. International Journal of Food Science. 2021;2021.

15. Deng Y, Xiong X, Liu X, He C, Guo S, Tang S, et al. Palygorskite combined probiotics improve the laying performance, hatching performance, egg quality, plasma antioxidative status, and immune response of broiler breeders. Italian Journal of Animal Science. 2021;20(1):1292-301.

16. Fathi M, Al-Homidan I, Al-Dokhail A, Ebeid T, Abou-Emera O, Alsagan A. Effects of dietary probiotic (Bacillus subtilis) supplementation on productive performance, immune response and egg quality characteristics in laying hens under high ambient temperature. Italian Journal of Animal Science. 2018;17(3):804-14.

17. Guo JR, Dong XF, Liu S, Tong JM. Effects of long-term Bacillus subtilis CGMCC 1.921 supplementation on performance, egg quality, and fecal and cecal microbiota of laying hens. Poult Sci. 2017;96(5):1280-9.

18. Martinez JS, Blount R, Park J, McIntyre D, Pavlidis H, Carey JJJoAPR. Effects of feeding original XPCTM to laying hens on egg production, component yield and composition. 2018;27(4):603-8.

19. Mazanko MS, Gorlov IF, Prazdnova EV, Makarenko MS, Usatov AV, Bren AB, et al. Bacillus Probiotic Supplementations Improve Laying Performance, Egg Quality, Hatching of Laying Hens, and Sperm Quality of Roosters. Probiotics and antimicrobial proteins. 2018;10(2):367-73.

20. Xiang Q, Wang C, Zhang H, Lai W, Wei H, Peng J. Effects of Different Probiotics on Laying Performance, Egg Quality, Oxidative Status, and Gut Health in Laying Hens. Animals (Basel). 2019;9(12).

21. Neijat M, Shirley RB, Barton J, Thiery P, Welsher A, Kiarie E. Effect of dietary supplementation of Bacillus subtilis DSM29784 on hen performance, egg quality indices, and apparent retention of dietary components in laying hens from 19 to 48 weeks of age. Poult Sci. 2019;98(11):5622-35.

22. Zhan HQ, Dong XY, Li LL, Zheng YX, Gong YJ, Zou XT. Effects of dietary supplementation with Clostridium butyricum on laying performance, egg quality, serum parameters, and cecal microflora of laying hens in the late phase of production. Poult Sci. 2019;98(2):896-903.

23. Mikulski D, Jankowski J, Mikulska M, Demey V. Effects of dietary probiotic (Pediococcus acidilactici) supplementation on productive performance, egg quality, and body composition in laying hens fed diets varying in energy density. Poult Sci. 2020;99(4):2275-85.

24. Liu X, Peng C, Qu X, Guo S, Chen JF, He C, et al. Effects of Bacillus subtilis C-3102 on production, hatching performance, egg quality, serum antioxidant capacity and immune response of laying breeders. J Anim Physiol Anim Nutr (Berl). 2019;103(1):182-90.

25. Zou X, Zhang M, Tu W, Zhang Q, Jin M, Fang R, et al. Bacillus subtilis inhibits intestinal inflammation and oxidative stress by regulating gut flora and related metabolites in laying hens. Animal. 2022;16(3):100474.

26. Zhou Y, Li S, Pang Q, Miao ZJP, proteins a. Bacillus amyloliquefaciens BLCC1-0238 can effectively improve laying performance and egg quality via enhancing immunity and regulating reproductive hormones of laying hens. 2020;12(1):246-52.

27. Macit M, Karaoglu M, Celebi S, Esenbuga N, Yoruk MA, Kaya A. Effects of supplementation of dietary humate, probiotic, and their combination on performance, egg quality, and yolk fatty acid composition of laying hens. Tropical Animal Health and Production. 2021;53(1):1-8.

28. Shi H, Zhang WL, Kim IH. Effects of dietary Bacillus subtilis RX7 and B2A supplementation on productive performance, egg quality, blood profiles, and excreta Salmonella counts in laying hens. Canadian Journal of Animal Science. 2020;100(3):411-7.

29. Song D, Wang Y, Lu Z, Wang W, Miao H, Zhou H, et al. Effects of dietary supplementation of microencapsulated Enterococcus fecalis and the extract of Camellia oleifera seed on laying performance, egg quality, serum biochemical parameters, and cecal microflora diversity in laying hens. 2019;98(7):2880-7.

30. Souza O, Adams C, Rodrigues B, Krause A, Bonamigo R, Zavarize K, et al. The Impact of Bacillus subtilis PB6 and Chromium Propionate on the Performance, Egg Quality and Nutrient Metabolizability of Layer Breeders. Animals. 2021;11(11):3084.

31. Upadhaya SD, Rudeaux F, Kim IH. Efficacy of dietary Bacillus subtilis and Bacillus licheniformis supplementation continuously in pullet and lay period on egg production, excreta microflora, and egg quality of Hyline-Brown birds. Poult Sci. 2019;98(10):4722-8.

32. Bhagwat Vishwanath G, Ellusamy B, Paramesh R, Nagalakshmi D, Srilatha T, Rao SVR. Effect of Supplementing Probiotic, Organic Acid and Herbal Extract (PhytoGrow) on Performance, Egg Quality and Gut Microbiota in White Leghorn Layers. Studies.10:11.

33. Wang W-w, Wang J, Zhang H-j, Wu S-g, Qi G-h. Effects of Clostridium butyricum on production performance and intestinal absorption function of laying hens in the late phase of production. Animal Feed Science and Technology. 2020;264:114476.

34. Khochamit N, Duangjinda M, Siripornadulsil S, Wongtangtintharn S, Siripornadulsil WJIJoAS. Effects of dried yeast, a byproduct of the brewery industry, on the egg production and quality and the immune response of laying hens. 2021;20(1):1135-46.

35. Zhang J-C, Chen P, Zhang C, Khalil MM, Zhang N-Y, Qi D-S, et al. Yeast culture promotes the production of aged laying hens by improving intestinal digestive enzyme activities and the intestinal health status. Poultry science. 2020;99(4):2026-32.

36. Darsi E, Zhaghari M. Effects of Bacillus subtilis PB6 supplementation on productive performance, egg quality and hatchability in broiler breeder hens under commercial farm condition. Journal of Applied Animal Research. 2021;49(1):109-17.

37. Uyanga VA, Jiao H, Zhao J, Wang X, Lin H. Dietary L-citrulline supplementation modulates nitric oxide synthesis and anti-oxidant status of laying hens during summer season. J Anim Sci Biotechnol. 2020;11:103.

38. Fascina VB, Pasquali GAM, Berto DA, Silva AdL, Garcia EA, Pezzato AC, et al. Effects of arginine and phytogenic additive supplementation on performance and health of brown-egg layers. Revista Brasileira de Zootecnia. 2017;46(6):502-14.

39. Neto MT, Dadalt J, Tse M. Dietary combination of chelated zinc and threonine and effects on egg production, egg quality and nutrient balance of Brown laying hens from 20 to 49 weeks of age. Animal Feed Science and Technology. 2020;267:114555.

40. Jiang S, El-Senousey HK, Fan Q, Lin X, Gou Z, Li L, et al. Effects of dietary threonine supplementation on productivity and expression of genes related to protein deposition and amino acid transportation in breeder hens of yellow-feathered chicken and their offspring. Poult Sci. 2019;98(12):6826-36.

41. Castro F, Kim Y, Xu H, Kim WJPS. The effect of total sulfur amino acid levels on growth performance and bone metabolism in pullets under heat stress. 2020;99(11):5783-91.

42. Parenteau IA, Stevenson M, Kiarie EGJPs. Egg production and quality responses to increasing isoleucine supplementation in Shaver white hens fed a low crude protein corn-soybean meal diet fortified with synthetic amino acids between 20 and 46 weeks of age. 2020;99(3):1444-53.

43. Joshi N, Wandita T, Yang S, Park H, Hwang SJTASJ. Effects of Supplementing Laying Hens with Purified Amino Acid Prepared from Animal Blood. 2019;42(1):46-52.

44. Jian H, Miao S, Liu Y, Li H, Zhou W, Wang X, et al. Effects of dietary valine levels on production performance, egg quality, antioxidant capacity, immunity, and intestinal amino acid absorption of laying hens during the peak lay period. Animals. 2021;11(7):1972.

45. Kim MC, Kim JH, Pitargue FM, Koo DY, Choi HS, Kil DY. Effect of dietary beta-mannanase on productive performance, egg quality, and utilization of dietary energy and nutrients in aged laying hens raised under hot climatic conditions. Asian-Australas J Anim Sci. 2017;30(10):1450-5.

46. Lei X, Lee K, Kim IJPs. Performance, egg quality, nutrient digestibility, and excreta microbiota shedding in laying hens fed corn-soybean-meal-wheat-based diets supplemented with xylanase. 2018;97(6):2071-7.

47. Nguyen H, Wu S-B, Bedford M, Nguyen X, Morgan NJBPS. Dietary soluble non-starch polysaccharide level and xylanase influence the gastrointestinal environment and nutrient utilisation in laying hens. 2021:1-11.

48. Bederska-Łojewska D, Arczewska-Włosek A, Świątkiewicz S, Orczewska-Dudek S, Schwarz T, Puchała M, et al. The effect of different dietary levels of hybrid rye and xylanase addition on the performance and egg quality in laying hens. British poultry science. 2019;60(4):423-30.

49. Sun HY, Kim IHJPs. Effects of multi-enzyme on production performance, egg quality, nutrient digestibility, and excreta noxious gas emission of early phase Hy-line brown hens. 2019;98(10):4889-95.

50. Habibollahi M, Abousadi MA, Nakhaee P. The effect of phytase on production performance, egg quality, calcium and phosphorus excretion, and fatty acids and cholesterol concentration in hy-line layers fed diets containing rice bran. Journal of Applied Poultry Research. 2019;28(3):688-98.

51. Arabshahi HA, Ghasemi HA, Hajkhodadadi I, Farahani AHK. Effects of multicarbohydrase and butyrate glycerides on productive performance, nutrient digestibility, gut morphology, and ileal microbiota in late-phase laying hens fed corn-or wheat-based diets. Poultry Science. 2021;100(5):101066.

52. Mu Y, Zhu LY, Yang A, Gao X, Zhang N, Sun L, et al. The effects of dietary cottonseed meal and oil supplementation on laying performance and egg quality of laying hens. Food Sci Nutr. 2019;7(7):2436-47.

53. Wang J, Kong F, Kim W. Effect of almond hulls on the performance, egg quality, nutrient digestibility, and body composition of laying hens. Poultry Science. 2021;100(9):101286.

54. Aguillón-Páez YJ, Romero LA, Diaz GJJAN. Effect of full-fat sunflower or flaxseed seeds dietary inclusion on performance, egg yolk fatty acid profile and egg quality in laying hens. 2020;6(2):179-84.

55. Mustafa A, Baurhoo B. Effect of feeding broccoli floret residues on leghorn layer performance and egg quality and nutrient digestibility. British poultry science. 2018;59(4):430-4.

56. Wang L, Li A, Shi J, Liu K, Cheng J, Song D, et al. Effects of different levels of cottonseed meal on laying performance, egg quality, intestinal immunity and hepatic histopathology in laying hens. Food and Agricultural Immunology. 2020;31(1):803-12.

57. Yuan N, Wang J, Ding X, Bai S, Zeng Q, Su Z, et al. Effects of supplementation with different rapeseed oil sources and levels on production performance, egg quality, and serum parameters in laying hens. 2019;98(4):1697-705.

58. Ghanima MMA, Alagawany M, Abd El-Hack ME, Taha A, Elnesr SS, Ajarem J, et al. Consequences of various housing systems and dietary supplementation of thymol, carvacrol, and euganol on performance, egg quality, blood chemistry, and antioxidant parameters. Poultry Science. 2020;99(9):4384-97.

59. Ding X, Yu Y, Su Z, Zhang K. Effects of essential oils on performance, egg quality, nutrient digestibility and yolk fatty acid profile in laying hens. Anim Nutr. 2017;3(2):127-31.

60. Dong X, Liu S, Tong J. Comparative effect of dietary soybean oil, fish oil, and coconut oil on performance, egg quality and some blood parameters in laying hens. Poultry science. 2018;97(7):2460-72.

61. Mousavi A, Mahdavi AH, Riasi A, Soltani-Ghombavani M. Synergetic effects of essential oils mixture improved egg quality traits, oxidative stability and liver health indices in laying hens fed fish oil. Animal Feed Science and Technology. 2017;234:162-72.

62. Areerob P, Dahlan W, Angkanaporn K. Dietary crude palm oil supplementation improves egg quality and modulates tissue and yolk vitamin E concentrations of laying hen. Animal Production Science. 2018;59(8):1491-500.

63. Muhammad AI, Mohamed DAA, Chwen LT, Akit H, Samsudin AA. Effect of sodium selenite, selenium yeast, and bacterial enriched protein on chicken egg yolk color, antioxidant profiles, and oxidative stability. Foods. 2021;10(4):871.

64. Chen J, Kuang Y, Qu X, Guo S, Kang K, He C. The effects and combinational effects of Bacillus subtilis and montmorillonite supplementation on performance, egg quality, oxidation status, and immune response in laying hens. Livestock Science. 2019;227:114-9.

65. Yalçın S, Eser H, Onbaşılar İ, Yalçın S, Karakaş Oğuz F. Effects of dietary sepiolite on performance, egg quality and some blood parameters in laying hens. 2016.

66. Yu Q, Liu H, Yang K, Tang X, Chen S, Ajuwon KM, et al. Effect of the level and source of supplementary dietary zinc on egg production, quality, and zinc content and on serum antioxidant parameters and zinc concentration in laying hens. 2020;99(11):6233-8.

67. Liu T, Li C, Li Y, Feng F. Glycerol Monolaurate Enhances Reproductive Performance, Egg Quality and Albumen Amino Acids Composition in Aged Hens with Gut Microbiota Alternation. Agriculture. 2020;10(7).

68. Gou Z, Fan Q, Li L, Wang Y, Lin X, Cui X, et al. High dietary copper induces oxidative stress and leads to decreased egg quality and reproductive performance of Chinese Yellow broiler breeder hens. Poultry Science. 2021;100(3):100779.

69. Lu J, Qu L, Ma M, Li YF, Wang XG, Yang Z, et al. Efficacy evaluation of selenium-enriched yeast in laying hens: effects on performance, egg quality, organ development, and selenium deposition. Poult Sci. 2020;99(11):6267-77.

70. Liu H, Yu Q, Fang C, Chen S, Tang X, Ajuwon KM, et al. Effect of selenium source and level on performance, egg quality, egg selenium content, and serum biochemical parameters in laying hens. Foods. 2020;9(1):68.

71. Han XJ, Qin P, Li WX, Ma QG, Ji C, Zhang JY, et al. Effect of sodium selenite and selenium yeast on performance, egg quality, antioxidant capacity, and selenium deposition of laying hens. Poult Sci. 2017;96(11):3973-80.

72. Xie C, Elwan HAM, Elnesr SS, Dong XY, Zou XT. Effect of iron glycine chelate supplementation on egg quality and egg iron enrichment in laying hens. Poult Sci. 2019;98(12):7101-9.

73. Miao LP, Li LL, Zhu MK, Dong XY, Elwan HAM, Zou XT. Excess dietary fluoride affects laying performance, egg quality, tissue retention, serum biochemical indices, and reproductive hormones of laying hens. Poult Sci. 2019;98(12):6873-9.

74. Li LL, Gong YJ, Zhan HQ, Zheng YX, Zou XT. Effects of dietary Zn-methionine supplementation on the laying performance, egg quality, antioxidant capacity, and serum parameters of laying hens. Poult Sci. 2019;98(2):923-31.

75. Abd El-Hack ME, Alagawany M, Amer SA, Arif M, Wahdan KMM, El-Kholy MS. Effect of dietary supplementation of organic zinc on laying performance, egg quality and some biochemical parameters of laying hens. J Anim Physiol Anim Nutr (Berl). 2018;102(2):e542-e9.

76. Cui Y-m, Zhang H-j, Zhou J-m, Wu S-g, Zhang C, Qi G-h, et al. Effects of long-term supplementation with amino acid-complexed manganese on performance, egg quality, blood biochemistry and organ histopathology in laying hens. Animal Feed Science and Technology. 2019;254.

77. Wang XC, Wang XH, Wang J, Wang H, Zhang HJ, Wu SG, et al. Dietary tea polyphenol supplementation improved egg production performance, albumen quality, and magnum morphology of Hy-Line Brown hens during the late laying period. J Anim Sci. 2018;96(1):225-35.

78. Zhang J, Zhang M, Liang W, Geng Z, Chen X. Green tea powder supplementation increased viscosity and decreased lysozyme activity of egg white during storage of eggs from Huainan partridge chicken. Italian Journal of Animal Science. 2020;19(1):586-92.

79. Zhou L, Ding X, Wang J, Bai S, Zeng Q, Su Z, et al. Tea polyphenols increase the antioxidant status of laying hens fed diets with different levels of ageing corn. Animal Nutrition. 2021;7(3):650-60.

80. Wang J, Jia R, Celi P, Ding X, Bai S, Zeng Q, et al. Green tea polyphenol epigallocatechin-3-gallate improves the antioxidant capacity of eggs. Food Funct. 2020;11(1):534-43.

81. Ding X, Du J, Zhang K, Bai S, Zeng Q, Peng H, et al. Tandem mass tag-based quantitative proteomics analysis and gelling properties in egg albumen of laying hens feeding tea polyphenols. Poultry Science. 2020;99(1):430-40.

82. Zhu YF, Wang JP, Ding XM, Bai SP, Qi SRN, Zeng QF, et al. Effect of different tea polyphenol products on egg production performance, egg quality and antioxidative status of laying hens. Animal Feed Science and Technology. 2020;267.

83. Xia B, Liu Y, Sun D, Liu J, Zhu Y, Lu L. Effects of green tea powder supplementation on egg production and egg quality in laying hens. Journal of Applied Animal Research. 2018;46(1):927-31.

84. Kaya H, Çelebi Ş, Kaya A, Gül M. Use of water-treated black tea waste instead of wheat bran in laying hen diets. Revista Brasileira de Zootecnia. 2018;47(0).

85. Wen C, Gu Y, Tao Z, Cheng Z, Wang T, Zhou Y. Effects of Ginger Extract on Laying Performance, Egg Quality, and Antioxidant Status of Laying Hens. Animals (Basel). 2019;9(11).

86. Damaziak K, Riedel J, Gozdowski D, Niemiec J, Siennicka A, Rog DJAoWUoLS-SAS. Effects of ginger or ginger and thyme extract in laying hens feeding on productive results and eggs quality. 2018;57.

87. Omer HAA, Ahmed SM, Abdel-Magid SS, El-Mallah GMH, Bakr AA, Abdel Fattah MM. Nutritional impact of inclusion of garlic (Allium sativum) and/or onion (Allium cepa L.) powder in laying hens’ diets on their performance, egg quality, and some blood constituents. Bulletin of the National Research Centre. 2019;43(1).

88. Kara K, Kocaoğlu Güçlü B, Baytok E, Şentürk M. Effects of grape pomace supplementation to laying hen diet on performance, egg quality, egg lipid peroxidation and some biochemical parameters. Journal of Applied Animal Research. 2015;44(1):303-10.

89. Vlaicu PA, Panaite TD, Turcu RP. Enriching laying hens eggs by feeding diets with different fatty acid composition and antioxidants. Scientific Reports. 2021;11(1):1-12.

90. Galli GM, Da Silva AS, Biazus AH, Reis JH, Boiago MM, Topazio JP, et al. Feed addition of curcumin to laying hens showed anticoccidial effect, and improved egg quality and animal health. Res Vet Sci. 2018;118:101-6.

91. Gumus H, Oguz MN, Bugdayci KE, Oguz FK. Effects of sumac and turmeric as feed additives on performance, egg quality traits, and blood parameters of laying hens. Revista Brasileira de Zootecnia. 2018;47(0).

92. Sharma MK, Dinh T, Adhikari PA. Production performance, egg quality, and small intestine histomorphology of the laying hens supplemented with phytogenic feed additive. Journal of Applied Poultry Research. 2020;29(2):362-71.

93. Galamatis D, Papadopoulos GA, Lazari D, Fletouris D, Petridou E, Arsenos GI, et al. Effects of Dietary Supplementation of Salvia officinalis L. in Organic Laying Hens on Egg Quality, Yolk Oxidative Stability and Eggshell Microbiological Counts. Animals. 2021;11(9):2502.

94. dos Santos AF, Da Silva AS, Galli GM, Paglia EB, Dacoreggio MV, Kempka AP, et al. Addition of yellow strawberry guava leaf extract in the diet of laying hens had antimicrobial and antioxidant effect capable of improving egg quality. 2020;29:101788.

95. Dilawar MA, Mun HS, Rathnayake D, Yang EJ, Seo YS, Park HS, et al. Egg quality parameters, production performance and immunity of laying hens supplemented with plant extracts. Animals. 2021;11(4):975.

96. Zheng M, Mao P, Tian X, Guo Q, Meng LJPs. Effects of dietary supplementation of alfalfa meal on growth performance, carcass characteristics, meat and egg quality, and intestinal microbiota in Beijing-you chicken. 2019;98(5):2250-9.

97. Vlaicu PA, Panaite TDJAB. Effect of dietary pumpkin (Cucurbita moschata) seed meal on layer performance and egg quality characteristics. 2021.

98. Sosnówka-Czajka E, Skomorucha I. Effect of supplementation with dried fruit pomace on the performance, egg quality, white blood cells, and lymphatic organs in laying hens. Poultry Science. 2021;100(9):101278.

99. Guo S, Lei J, Liu L, Qu X, Li P, Liu X, et al. Effects of Macleaya cordata extract on laying performance, egg quality, and serum indices in Xuefeng black-bone chicken. Poultry science. 2021;100(4):101031.

100. Fernandes RTV, Gonçalves AA, Arruda AMVd. Production, egg quality, and intestinal morphometry of laying hens fed marine microalga. Revista Brasileira de Zootecnia. 2020;49.

101. Selim S, Hussein E, Abou-Elkhair R. Effect of Spirulina platensis as a feed additive on laying performance, egg quality and hepatoprotective activity of laying hens2018.

102. Shi HT, Wang BY, Bian CZ, Han YQ, Qiao HX. Fermented Astragalus in diet improved laying performance, egg quality, antioxidant and immunological status and intestinal microbiota in laying hens. AMB Express. 2020;10(1):159.

103. Moon SG, Lee SK, Lee WD, Niu KM, Hwang WU, Oh JS, et al. Effect of dietary supplementation of a phytogenic blend containing Schisandra chinensis, Pinus densiflora, and Allium tuberosum on productivity, egg quality, and health parameters in laying hens. Anim Biosci. 2021;34(2):285-94.

104. Fan GJ, Shih BL, Lin HC, Lee TT, Lee CF, Lin YF. Effect of dietary supplementation of Sargassum meal on laying performance and egg quality of Leghorn layers. Anim Biosci. 2021;34(3):449-56.

105. Wan Y, Ma R, Qi R, Li Y, Liu W, Li J, et al. Dietary fresh lemon improves the albumen quality, immune status and lipid metabolism of Jingfen laying hens during the late laying period. 2021;20(1):834-41.

106. Li XL, He WL, Yang ML, Yan YM, Xue YH, Zhao ST. Effect of dietary supplementation of Ligustrum lucidum on performance, egg quality and blood biochemical parameters of Hy-Line Brown hens during the late laying period. Animal. 2017;11(11):1899-904.

107. Chen X, Zhang Y, Ma W, Wang Z. Effects of Ligustrum lucidum on egg production, egg quality, and caecal microbiota of hens during the late laying period. Italian Journal of Animal Science. 2020;19(1):687-96.

108. Xie T, Bai SP, Zhang KY, Ding XM, Wang JP, Zeng QF, et al. Effects of Lonicera confusa and Astragali Radix extracts supplementation on egg production performance, egg quality, sensory evaluation, and antioxidative parameters of laying hens during the late laying period. Poult Sci. 2019;98(10):4838-47.

109. Feng Z, Gong J, Zhao G, Lin X, Liu Y, Ma KJBps. Effects of dietary supplementation of resveratrol on performance, egg quality, yolk cholesterol and antioxidant enzyme activity of laying hens. 2017;58(5):544-9.

110. Gong H, Yang Z, Celi P, Yan L, Ding X, Bai S, et al. Effect of benzoic acid on production performance, egg quality, intestinal morphology, and cecal microbial community of laying hens. Poult Sci. 2021;100(1):196-205.

111. Dansou DM, Wang H, Nugroho RD, He W, Zhao Q, Zhang JJA. Assessment of Response to Moderate and High Dose Supplementation of Astaxanthin in Laying Hens. 2021;11(4):1138.

112. Heng N, Gao S, Guo Y, Chen Y, Wang L, Sheng X, et al. Effects of supplementing natural astaxanthin from Haematococcus pluvialis to laying hens on egg quality during storage at 4° C and 25° C. 2020;99(12):6877-83.

113. Chen F, Zhang H, Du E, Jin F, Zheng C, Fan Q, et al. Effects of magnolol on egg production, egg quality, antioxidant capacity, and intestinal health of laying hens in the late phase of the laying cycle. 2021;100(2):835-43.

114. Zhu A, Zhang K, Wang J, Bai S, Zeng Q, Peng H, et al. Effect of different concentrations of neohesperidin dihydrochalcone on performance, egg quality, serum biochemistry and intestinal morphology in laying hens. 2021;100(7):101097.

115. Zhang L, Zhong G, Gu W, Yin N, Chen L, Shi S. Dietary supplementation with daidzein and Chinese herbs, independently and combined, improves laying performance, egg quality and plasma hormone levels of post-peak laying hens. Poultry Science. 2021;100(6):101115.

116. Chen X, Zhan Y, Ma W, Zhu Y, Wang ZJASJ. Effects of Antimicrobial peptides on egg production, egg quality and caecal microbiota of hens during the late laying period. 2020;91(1):e13387.

117. Chang X, Qiu K, Wang J, Zhang H, You S, Mi S, et al. The Evaluation of UPro as a New Nutrient on High-Quality Egg Production From the Perspective of Egg Properties, Intestinal Histomorphology, and Oviduct Function of Laying Hens. 2021;8.

118. İskender H, Yenice G, Dokumacioglu E, Kaynar O, Hayirli A, Kaya AJBps. Comparison of the effects of dietary supplementation of flavonoids on laying hen performance, egg quality and egg nutrient profile. 2017;58(5):550-6.

119. Adli DN. The effect of replacing fish meal with Sago larvae meal (SLM) on egg production and quality of laying hens. Livestock Research for Rural Development. 2021;33.

120. Patterson P, Acar N, Ferguson A, Trimble L, Sciubba H, Koutsos E. The impact of dietary Black Soldier Fly larvae oil and meal on laying hen performance and egg quality. Poultry science. 2021;100(8):101272.

121. Abd El-Hack ME, Alagawany M, Mahrose KM, Arif M, Saeed M, Arain MA, et al. Productive performance, egg quality, hematological parameters and serum chemistry of laying hens fed diets supplemented with certain fat-soluble vitamins, individually or combined, during summer season. Animal Nutrition. 2019;5(1):49-55.

122. Wang J, Qiu L, Gong H, Celi P, Yan L, Ding X, et al. Effect of dietary 25-hydroxycholecalciferol supplementation and high stocking density on performance, egg quality, and tibia quality in laying hens. Poultry Science. 2020;99(5):2608-15.

123. Chen C, Turner B, Applegate TJ, Litta G, Kim WK. Role of long-term supplementation of 25-hydroxyvitamin D3 on egg production and egg quality of laying hen. Poult Sci. 2020;99(12):6899-906.
